# Isomeric dibenzooctazethrene diradicals for high-performance air-stable organic field-effect transistors†

**DOI:** 10.1039/d2sc03667c

**Published:** 2022-09-13

**Authors:** Chaoyang Zong, Shuyuan Yang, Yajing Sun, Lifeng Zhang, Jinlian Hu, Wenping Hu, Rongjin Li, Zhe Sun

**Affiliations:** Institute of Molecular Plus, Department of Chemistry, Haihe Laboratory of Sustainable Chemical Transformations, Tianjin University 92 Weijin Road Tianjin 300072 China zhesun@tju.edu.cn; Tianjin Key Laboratory of Molecular Optoelectronic Sciences, Department of Chemistry, School of Science, Tianjin University 92 Weijin Road Tianjin 300072 China lirj@tju.edu.cn

## Abstract

Realizing both high-performance and air-stability is key to advancing singlet-diradical-based semiconductors to practical applications and realizing their material potential associated with their open-shell nature. Here a concise synthetic route toward two stable dibenzooctazethrene isomers, DBOZ1 and DBOZ2, was demonstrated. In the crystalline phase, DBOZ2 exhibits two-dimensional brick wall packing with a high degree of intermolecular electronic coupling, leading to a record-breaking hole mobility of 3.5 cm^2^ V^−1^ s^−1^ for singlet diradical transistors, while retaining good device stability in the ambient air.

## Abstract 2

Realizing both high-performance and air-stability is key to advancing singlet-diradical-based semiconductors to practical applications and realizing their material potential associated with their open-shell nature.

## Graphical abstract

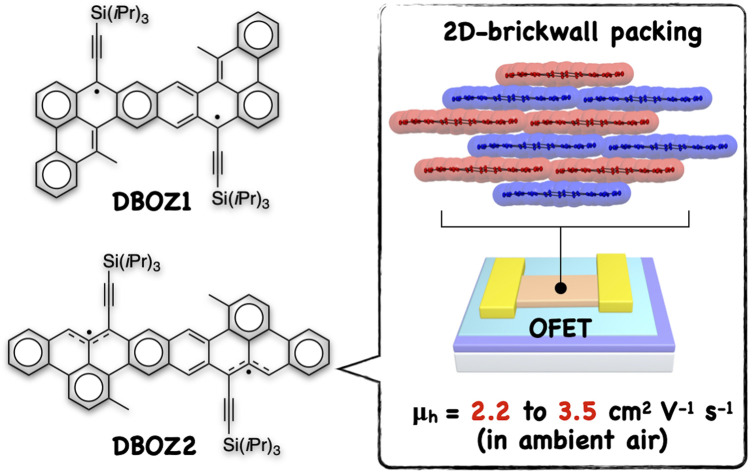

## Introduction

Organic semiconductors with high charge carrier mobilities are highly desirable for the modern technologies such as integrated circuits,^1^ displays,^2^ storage devices,^3^ and sensors,^4^ due to their low fabrication cost, mechanical flexibility and structural versatility.^5^ The development of new organic materials is typically the foundation of innovation in organic semiconductors. Over the past decades, singlet diradicals have emerged as promising candidates for organic field-effect transistors (OFETs), because the intermolecular spin–spin interaction could promote efficient orbital overlap in the solid state,^6^ which increases the transfer integral that facilitates charge transport.^7^ Additionally, singlet diradicals generally exhibit redox amphoterism.^8^ Both of the positively and negatively charged species could be stabilized by recovering aromaticity from the pro-aromatic quinoidal neutral species,^9^ which holds promise for ambipolar transistors.^10^ Since the seminal work of Chikamatsu *et al.* for the first fabrication of diradical-OFET^11^ with a bis(phenalenyl) system synthesized by Kubo *et al.*,^12^ singlet-diradical-based transistors have been reported on molecular systems including indenofluorene analogues,^13^ zethrenes,^14^ peri-acenoacenes,^15^ and others.^16^ So far, the highest hole mobility was reported by Frigoli *et al.* as 1.4 cm^2^ V^−1^ s^−1^,^15*c*^ measured under a N_2_ atmosphere. Hampered by the intrinsic material instability, most of the reported transistors were fabricated and operated under inert atmosphere, which limited their practical applications. Therefore, the further development of singlet diradical semiconductors hinges on achieving materials with both air-stability and high-performance.

**Fig. 1:**
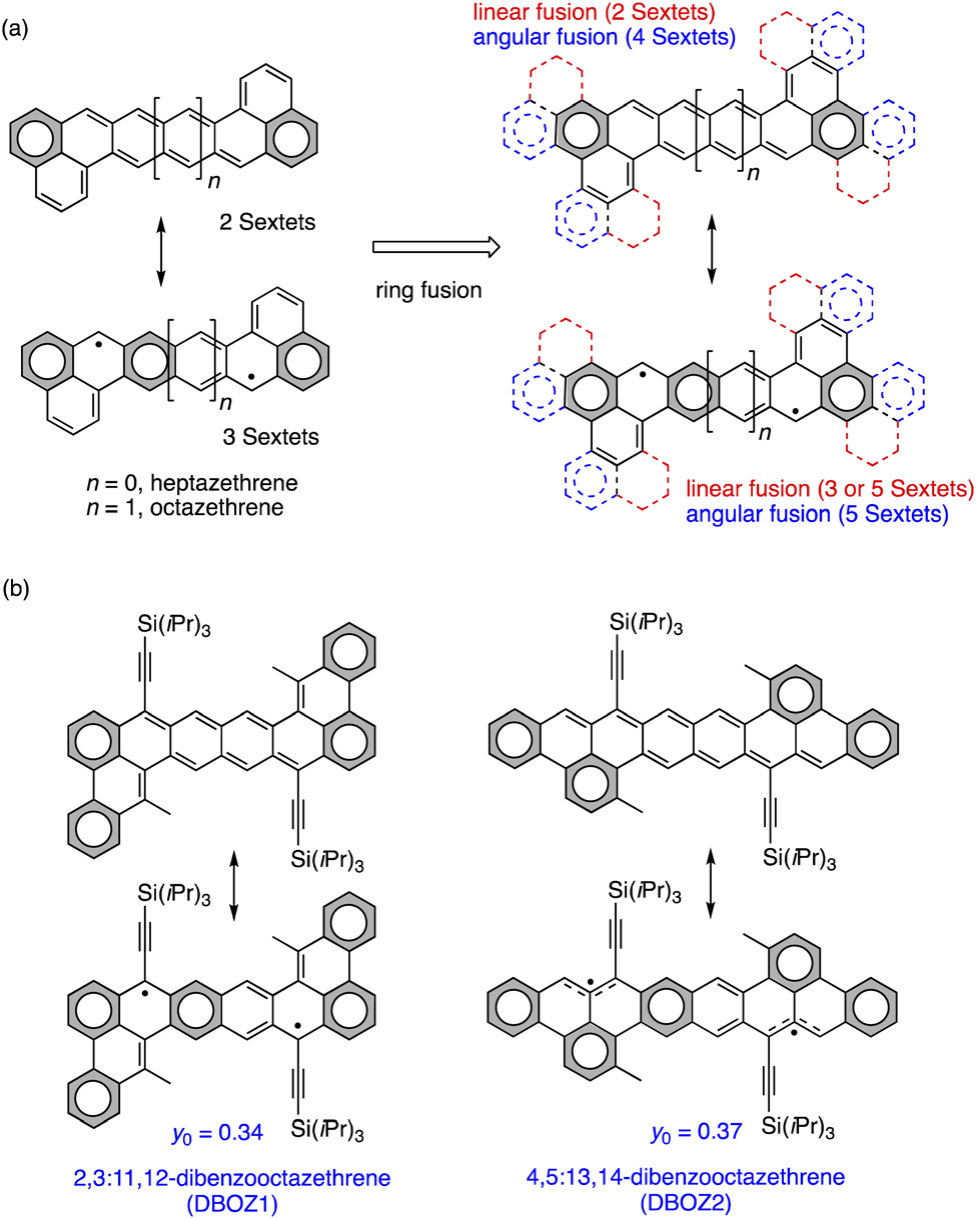
(a) Molecular design for stable benzoannulated heptazethrene and octazethrene. (b) Resonance structures of DBOZ1 and DBOZ2 in this study and their diradical index (*y*_0_), *y*_0_ was calculated from Yamaguchi Scheme.

Unfortunately, the stability and diradical-related properties are often paradoxical, as increasing the diradical character would usually sacrifice the material stability. For the open-shell molecules, the most common decomposition pathways are oxidation^17^ and oligomerization/polymerization.^18^ The former is due to the presence of singly occupied molecular orbital (SOMO) and the reactive nature of unpaired electrons, which is associated with the thermodynamic stability.^19^ The latter is due to radical–radical coupling pathways associated with the kinetic stability. To increase the thermodynamic stability, a molecular design based on tuning the ring fusion mode was proposed for extended zethrenes. As shown in Fig. 1a, benzenoid rings could be fused to the terminal naphthalene subunits of heptazethrene or octazethrene either linearly or angularly. The angular fusion would add two more Clar Sextets to both the closed-shell structure and the open-shell structure, as opposed to the linear fusion mode, which could only add more Clar Sextet rings to the open-shell resonance structure. Therefore, it would be reasonable to expect that material stability would improve. Stable dibenzoheptazethrene (DBHZ) derivatives and fabricated OFET devices that could operate under ambient air have previously been prepared using this design concept. However, the hole mobility was just 0.15 cm^2^ V^−1^ s^−1^. A major reason for the unsatisfactory device performance was the lack of the electronic coupling in the crystalline state, which may result from the small diradical character of dibenzoheptazethrene isomers (*y*_0_ = 0.11–0.13).^20,21^

In this work, the synthesis of π-extended dibenzooctazethrene (DBOZ), a hitherto elusive member of the zethrene family,^22^ is reported. Two constitutional isomers of DBOZ, 2,3:11,12-dibenzooctazethrene (DBOZ1) and 4,5:13,14-dibenzooctazethrene (DBOZ2) with enhanced diradical character (*y*_0_ = 0.34 and 0.37, respectively, Fig. 1b) are designed. It was envisioned that the extension of the conjugation and the enhancement of the diradical character would further increase the intermolecular electronic coupling, and would also help reduce the reorganization energy.^23^ The bulky triisopropylsilylethynyl (TIPSe) groups were introduced to the most spin-residing positions to kinetically block the reactive sites. Gratifyingly, DBOZ2 exhibits a record-high hole mobility of 3.5 cm^2^ V^−1^ s^−1^ among singlet diradicals (Fig. S18 and Table S6†), while retaining good device stability in air.

## Results and discussion

**Scheme 1:**
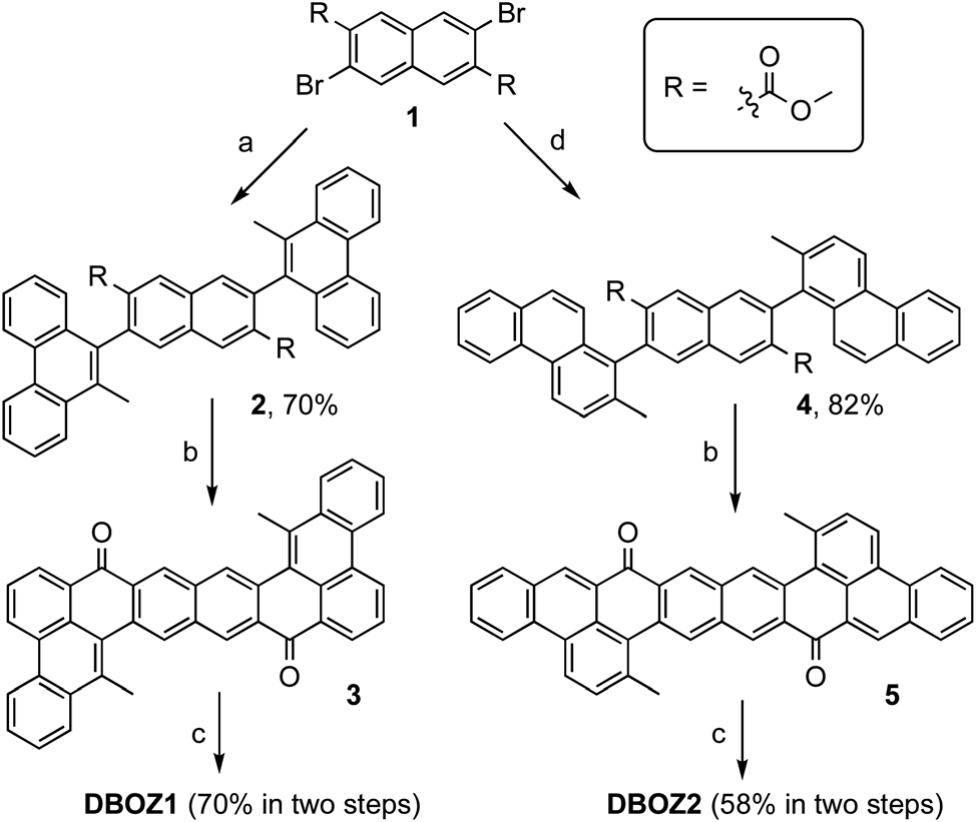
Synthesis of DBOZ1 and DBOZ2. Reagents and conditions: (a) (10-methylphenanthren-9-yl)boronic acid, Pd(PPh_3_)_4_, toluene/ethanol, Na_2_CO_3_ (aq), 90 °C, 60 h; (b) CH_3_SO_3_H, 100 °C, 24 h; (c) (1) *n*BuLi, triisopropylsilylacetylene, 2 h; (2) SnCl_2_, rt, 12 h; (d) (2-methylphenanthren-1-yl)boronic acid, Pd(PPh_3_)_4_, toluene/ethanol, Na_2_CO_3_ (aq), 90 °C, 60 h.

As shown in Scheme 1, the synthesis of both DBOZ1 and DBOZ2 started with the same precursor dimethyl 3,7-dibromonaphthalene-2,6-dicarboxylate (1). With pre-installed methyl groups to prevent the unwanted cyclization products, Suzuki coupling reaction between 1 and (10-methylphenanthren-9-yl)boronic acid/(2-methylphenanthren-1-yl)boronic acid afforded compounds 2/4 in 70% and 82% yields, respectively. Then, intramolecular Friedel–Crafts acylation produced diketone compounds 3/5, which further underwent nucleophilic addition with TIPSe lithium regent followed by reduction with SnCl_2_ to give the desired products. The structures of DBOZ1 and DBOZ2 were unequivocally determined by NMR spectroscopy, mass spectrometry, and X-ray crystallographic analysis (*vide infra*), and their purity were supported by HPLC analysis (Fig. S1 and S2†).

The solution phase stabilities of DBOZ1 and DBOZ2, especially toward oxidation, were investigated by tracking the spectral decay at the maxima absorptions in air-saturated toluene solutions (Fig. S4 and S5†). Half-life times (*τ*_1/2_) of 36 days and 8 days were obtained for DBOZ1 and DBOZ2, respectively, from the fitting of first-order kinetics.^24^ For comparison, the stability of the benchmark organic semiconductor, TIPSe-substituted pentacene,^25^ was measured under the same condition and showed *τ*_1/2_ as 3 days, suggesting that both DBOZ isomers were sufficiently stable for the device fabrication. The increased aromatic stabilization and kinetic protection at the strategic positions with high spin densities should be the source of the enhancement in air stability. The thermal stabilities were inspected by thermogravimetric analysis (TGA), and the decomposition temperatures (*T*_d_, corresponding to 5% weight loss) for DBOZ1 and DBOZ2 were estimated to be 280 °C and 326 °C, respectively (Fig. S6†). Together, both the air-stability in solution and the thermostability in the solid state suggests that they qualify as stable organic materials.

**Fig. 2:**
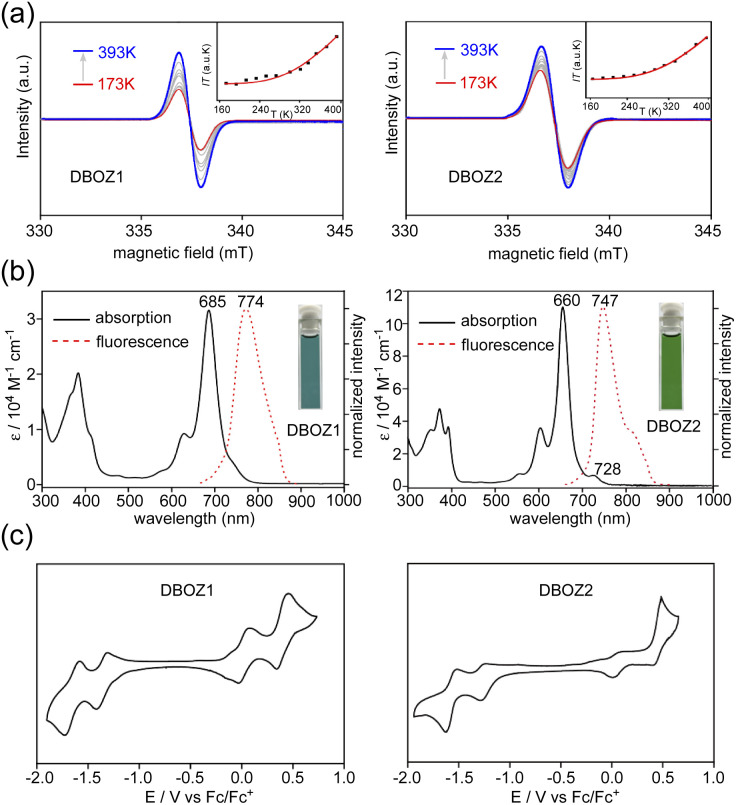
(A) VT-EPR spectra and IT-T curve fitting for DBOZ1 and DBOZ2. (b) UV-vis absorption and fluorescence spectra of DBOZ1 and DBOZ2 in toluene. Inset shows pictures of their toluene solutions. (c) Cyclic voltammograms of DBOZ1 and DBOZ2.

The DFT calculations at (U)CAM-B3LYP/6-31G(d,p) level predicted a singlet diradical ground state for both DBOZ1 and DBOZ2 (Table S5†). The solid samples of both DBOZ1 and DBOZ2 showed intense electron paramagnetic resonance (EPR) signals at 393 K, the intensities gradually decreasing upon cooling to 173 K, indicative of a diamagnetic singlet ground state with a thermally populated paramagnetic triplet state (Fig. 2a). The singlet-triplet energy gaps (Δ*E*_S–T_) were estimated as −3.35 kcal mol^−1^ and –3.37 kcal mol^−1^ for DBOZ1 and DBOZ2, respectively, by fitting of the Bleaney–Bowers equation.^26^ Small Δ*E*_S–T_ values were also evident in the solution phase, as a progressive line-broadening of ^1^H NMR spectra was observed for both DBOZ1 and DBOZ2, upon heating from 293 K to 373 K (Fig. S7 and S8†). This phenomenon was not observed for DBHZ isomers due to their larger Δ*E*_S–T_ values.^20^

Both isomers exhibited good solubility in toluene to give blue and green colored solutions. As shown in Fig. 2b, the absorption maximum (*λ*_max_) of DBOZ1 was located at 685 nm (*ε* = 33 679 M^−1^ cm^−1^), whereas DBOZ2 displayed a more prominent *λ*_max_ at 660 nm (*ε* = 110 400 M^−1^ cm^−1^). The signature shoulder peak at the lower energy region corresponding to the double-excitation^27^ of singlet diradicals was barely noticeable for DBOZ1, but was clearly observed for DBOZ2 at 728 nm. Weak emissions close to the near-infrared region were observed at 774 nm for DBOZ1 and 747 nm for DBOZ2 when excited at their absorption maximum, with fluorescence quantum yields of 8% and 13%, respectively. The slightly larger Stokes shift of DBOZ1 (1679 cm^−1^) than DBOZ2 (1765 cm^−1^) should result from its nonplanar and less rigid molecular skeleton (see crystal structures below).

Cyclic voltammetry (CV) measurements in dichloromethane revealed salient amphoteric redox behavior with reversible and nearly symmetric oxidation and reduction waves (Fig. 2c). The half-wave potentials for oxidation (*E*^ox^_1/2_) were −0.02 and 0.39 V for DBOZ1 and 0.02 and 0.45 V for DBOZ2, and the half-wave potentials for reduction (*E*^red^_1/2_) were −1.36 and −1.69 V for DBOZ1 and –1.38 and −1.66 V for DBOZ2. The HOMO/LUMO energies of DBOZ1 and DBOZ2 were estimated as −4.62/–3.57 eV and −4.65/–3.58 eV, respectively, providing narrow HOMO–LUMO gaps of 1.05 and 1.07 eV. Such narrow band gaps help balance the electron and hole injection barrier between the electrode and semiconductor to give ambipolar behavior.

**Fig. 3:**
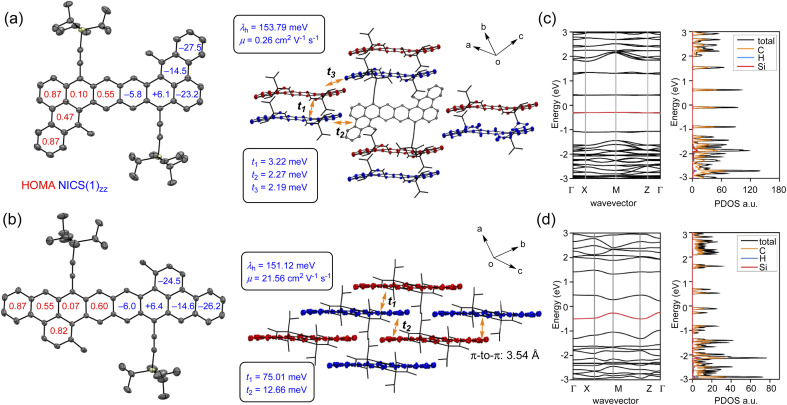
X-ray crystallographic structures and crystal packing for (a) DBOZ1 and (b) DBOZ2, and calculated band structure and density of states of (c) DBOZ1 and (d) DBOZ2. HOMA, NICS(1)_*zz*_, calculated charge carrier mobility, reorganization energy and transfer integrals were shown.

The crystal structures of DBOZ1 and DBOZ2 were determined by X-ray diffraction. As shown in Fig. 3a and b, the molecular skeleton of DBOZ2 is perfectly planar whereas DBOZ1 deviated from planarity. To visualize the localization of the Clar Sextet rings and to elucidate the electronic ground states, the harmonic oscillator model of aromaticity (HOMA)^28^ was performed, which indicated a prominent phenanthrene subunit at the terminal and naphthalene subunit in the middle for both DBOZ1 and DBOZ2, in line with the diradical canonical structures. The NICS(1)_*zz*_ calculations provided additional evidence in favor of these findings.^29^ More attractive features were illustrated from the packing structure: whereas DBOZ1 still adopted dimerized packing similar to the DBHZ isomers,^20^ DBOZ2 finally provided well-ordered 2D brick wall packing suitable for intermolecular charge transport, which was long-sought-after for extended zethrene diradicals (Fig. 3b). The average intermolecular π–π distance was around 3.54 Å. The drastic change in packing pattern should associate to the extension of conjugation in the linear direction, rather than the oblique direction, to afford an octacene-like structure.

The first-principle calculation was carried out to study their electronic structures and charge transport properties. In the hopping model, the charge carrier mobility (*μ*) is determined by the reorganization energy (*λ*) and the transfer integrals (*t*). The former is related to the structure relaxing effect within the charge transport process, measuring the strength of the electron–phonon interaction, and the latter represents the degree of electronic coupling between adjacent molecules.^30^ As shown in Fig. 3a and b, the values of *λ* and *t* were calculated at the B3LYP/6-31G(d) and PBE0/6-31G(d) levels, respectively. The *λ* values of DBOZ1 (153.79 meV) and DBOZ2 (151.12 meV) were similar, whereas the maximum *t* values were remarkably different (3.22 meV for DBOZ1 *vs.* 75.01 meV for DBOZ2), because of a more compact packing of DBOZ2. The hole mobilities were estimated using Fermi's golden rule and Monte Carlo simulation,^27^ to produce 0.26 cm^2^ V^−1^ s^−1^ for DBOZ1 and 21.56 cm^2^ V^−1^ s^−1^ for DBOZ2. The electronic band structures and the electronic density of states (DOS) calculated using the projector augmented wave (PAW) method and the Perdew−Burke−Ernzerhof (PBE) exchange–correlation^31^ functional also provided consistent results. The bandwidth of the valence band of DBOZ2 (266.67 meV) was much larger than that of DBOZ1 (14.43 meV, Fig. 3c and d). The dispersive valence band led to a smaller hole effective mass for DBOZ2 (0.051*m*_e_) compared to DBOZ1 (0.509*m*_e_) (see the ESI† for details).

**Fig. 4:**
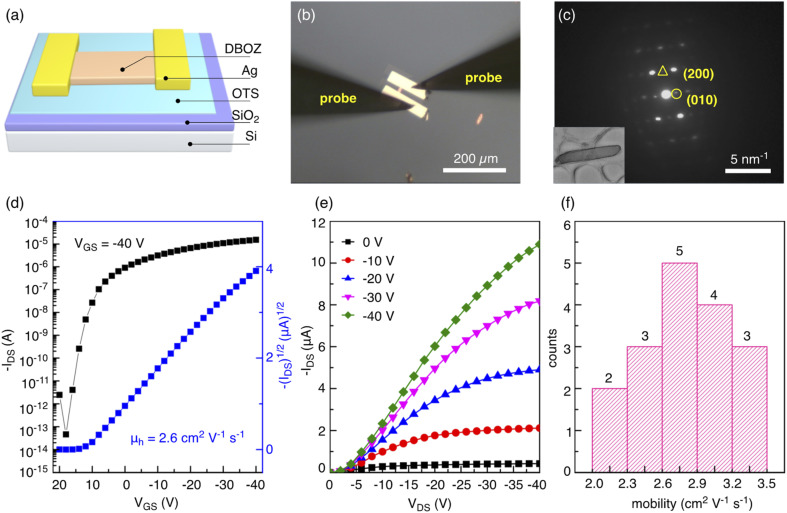
(a) Schematic diagram of the OFET. (b) OM images of an OFET based on a single crystal of DBOZ2. (c) The SAED pattern of a single crystal of DBOZ2. The inset shows the corresponding TEM image of the crystal (length of 7.6 μm). (d, e) Typical transfer and output curves of the OFETs based on single crystals of DBOZ2 in air (channel length = 16.4 μm, channel width = 8.4 μm). (f) The mobility distribution of 17 devices of DBOZ2.

Encouraged by the dense packing of DBOZ2 and the calculation results, single crystalline organic field-effect transistors (OFETs) were constructed to probe their charge transport properties (Fig. 4a and b). Crystals of both isomers were grown on silicon substrates with 300 nm SiO_2_ by drop casting (see the ESI† for details). The optical microscopy (OM, Fig. S9†) and atomic force microscopy images (AFM, Fig. S10†) of DBOZ1 and DBOZ2 crystals showed regular morphologies. Under a polarized optical microscopy (POM), the colors of both crystals changed uniformly when the substrate was rotated by 45°, indicating the absence of any grain boundaries and the single crystalline nature (Fig. S9†). The bright-field transmission electronic microscopy (TEM) images and the corresponding selected area electron diffraction (SAED) patterns of the crystals (Fig. 4c and S12†) further indicated that they were single crystals. Bottom-gate top-contact OFETs based on single crystals of DBOZ1 and DBOZ2 were constructed and their charge transport properties were measured in ambient air (Fig. 4d, e and S11, S12†). The maximum hole mobilities of DBOZ1 and DBOZ2 were 0.0085 cm^2^ V^−1^ s^−1^ and 3.5 cm^2^ V^−1^ s^−1^, respectively. It was noted that the value of 3.5 cm^2^ V^−1^ s^−1^ represents a record among singlet diradicals (Table S6†).^15*c*^ The mobility distribution of 17 devices of DBOZ2 is shown in Fig. 4f, by which the average mobility was calculated to be 2.8 cm^2^ V^−1^ s^−1^. The OFETs based on DBOZ2 showed high mobility and high stability. The bias stress stability test exhibited almost identical transfer curves in 10 cycles in ambient air. After the device being stored in ambient air for over one month, negligible degradation was observed (Fig. S15†). The device stability is comparable to that of organic semiconductors with a closed-shell ground state.^32^

## Conclusions

In summary, we have described a concise synthesis of two stable isomeric dibenzooctazethrene derivatives, DBOZ1 and DBOZ2, with singlet diradical ground states. Benefited from the extension of π-conjugation and the enhancement of the diradical character, 2D-brick wall packing and efficient intermolecular electronic coupling in the solid state were achieved for DBOZ2, leading to a record-high hole mobility of up to 3.5 cm^2^ V^−1^ s^−1^ for singlet diradical semiconductors, which outperformed the amorphous silicon.^33^ Additionally, the device was fabricated and operated in ambient air with good bias-stress stability and storage stability, demonstrating the practicality of singlet-diradical-based semiconductors in the field of organic electronics. Once properly stabilized and functionalized, singlet diradicals could be a valuable addition to the library of organic semiconductors.

## Data availability

The ESI† contains detailed description for the synthetic method, computational method and device fabrications. The supplementary spectroscopic and crystallographic data were also provided.

## Author contributions

Z. S. and R. L. supervised the project. C. Z. and J. H. performed synthetic experiments. S. Y. performed device fabrication and characterization. Y. S. and C. Z. performed theoretical calculations. L. Z. performed TEM measurements. All authors analyzed the data, discussed the results, and contributed to the manuscript writing.

## Conflicts of interest

There are no conflicts to declare

## Supplementary Material

SC-013-D2SC03667C-s001

SC-013-D2SC03667C-s002
