# Supplementary material for: Isomeric dibenzooctazethrene diradicals for high-performance air-stable organic field-effect transistors
Source: Chem Sci. 2022 Sep 13;13(38):11442–7. doi: 10.1039/d2sc03667c (PMC9533412; doi:10.1039/d2sc03667c)
Supplement: SC-013-D2SC03667C-s001 [file SC-013-D2SC03667C-s001.pdf]

Electronic Supplementary Material (ESI) for Chemical Science.
This journal is © The Royal Society of Chemistry 2022

Supporting Information

Isomeric Dibenzooctazethrene Diradicals for High-Performance Air-Stable Organic Field-Effect Transistors

Chaoyang Zong,[1†] Shuyuan Yang,[2†] Yajing Sun,[2] Lifeng Zhang,[1] Jinlian Hu,[1] Wenping Hu,[2] Rongjin Li,*[2] Zhe Sun*[1]

[1] Institute of Molecular Plus, Department of Chemistry and Haihe Laboratory of Sustainable Chemical Transformations, Tianjin University, 92 Weijin Road, Tianjin 300072

[2] Tianjin Key Laboratory of Molecular Optoelectronic Sciences, Department of Chemistry, School of Science, Tianjin University, 92 Weijin Road, Tianjin 300072

*Corresponding Author(s): lirj@tju.edu.cn; zhesun@tju.edu.cn; †C. Zong and S. Yang contributed equally to this work.

Table of contents

1. Experimental section S2
2. Additional spectra and charts S7
3. X-ray crystallographic data S11
4. Theoretical calculations S13
5. OFET fabrication and characterization S17
6. $^{1}H/^{13}C$NMR and MS spectra for new compounds S23
7. References S28

1. Experimental section

1.1 General

All air-sensitive reactions were carried out under argon atmosphere using standard Schlenk technique. For moisture sensitive reactions, Anhydrous THF and toluene were distilled with sodium under argon prior to use. For column chromatography, silica gel (200-300 mesh) was used. All other reagents were purchased from the commercial source and used without further purification. Dimethyl 3,7-dibromonaphthalene-2,6-dicarboxylate (**1**)[1], (10-methylphenanthren-9-yl)boronic acid (**6**) and (2-methylphenanthren-1-yl)boronic acid (**7**)[2] were prepared according to literature method. Proton (^{1}H) NMR and carbon (^{13}C) NMR spectra were recorded on JNM-ECZ600R/S1 with tetramethylsilane (TMS) as the internal standard. Chemical shifts were given in ppm relative to residue protons ($CDCl_3$: δ 7.26 for ^{1}H, δ 77.16 for ^{13}C; DMSO-d_6: δ 2.50 for ^{1}H, δ 39.52 for ^{13}C;1,1,2,2-$C_2D_2Cl_4$: δ 6.00 for ^{1}H, δ 73.78 for ^{13}C). High resolution Electrospray Ionization (ESI) mass spectra were performed on Bruker Micro TOF-QII instrument. Analytical high pressure liquid chromatography (HPLC) was performed with SHIMADZU LC-2030C 3D Plus equipment. UV-vis absorption spectra were recorded on SHIMADZU UV-2600 spectrophotometer. The electrochemical measurements were carried out in anhydrous DCM containing $^{n}BuNPF_6$ as supporting electrolyte under argon atmosphere by CHI 620E electrochemical analyzer. A three-electrode system with glassy carbon as working electrode, $Ag/AgNO_3$ as reference electrode, Pt wire as counter electrode was applied. The potential was calibrated against Ferrocene/Ferrocenium couple. The diffraction data were collected on XtaLAB Synergy diffractometer equipped with Hypix6000HE detector using a single-wavelength X-ray source. The ESR spectra were obtained on a JES-FA200 spectrometer. The VT ESR data in toluene solution were fitted with Bleaney-Bowers equation:

$$IT = \frac{C}{k_B[3 + \exp(-2J/k_BT)]}$$

Where, I is the ESR intensity, C is a constant, k_B is Boltzmann constant, and $-2J$ is correlated to the energy gap between the singlet ground state and the triplet excite state. Optical microscope (OM) and polarized optical microscopy (POM) images were obtained by Nikon ECLIPSE Ci-POL polarized optical microscopes. Powder XRD

measurements were performed by a Rigaku Smartlab diffractometer at 45 kV and 200 mA with monochromatic Cu Kα radiation. Transmission electron microscope (TEM) and selected area electron diffraction (SAED) measurements were conducted on a Tecnai G2 F20 transmission electron microscope. The OFETs were measured using a Keithley 4200 SCS in ambient environment and room temperature.

Synthesis of Compound 2

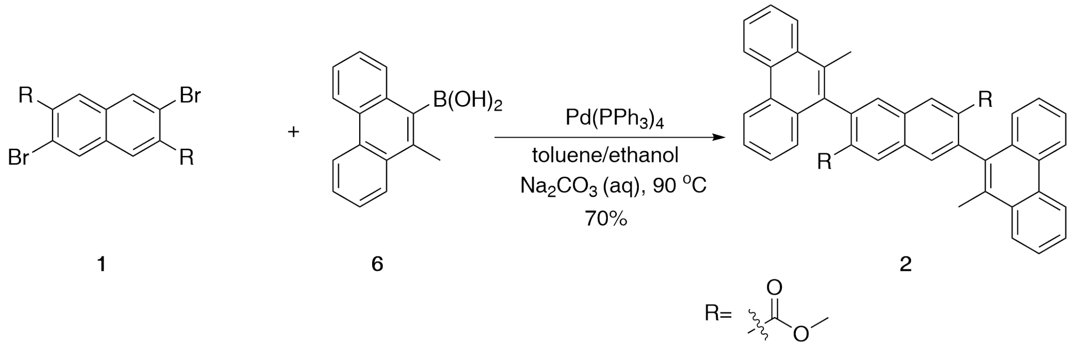

A mixture of dimethyl 3,7-dibromonaphthalene-2,6-dicarboxylate **1** (80 mg, 0.20 mmol), (10-methylphenanthren-9-yl) boronic acid **6** (236 mg, 1.0 mmol), $Pd(PPh_3)_4$ (24 mg, 0.02 mmol), toluene (2.5 mL), ethanol (0.3 mL), and Na_2CO_3 aqueous solution (2 M, 0.4 mL) were added to a 25 mL Schlenk flask and the mixture was further degassed by three freeze-pump-thaw cycles. The reaction was then heated at 90 °C for 60 hours under argon atmosphere. After cooling to the room temperature, the mixture was poured into water and extracted with DCM. The organic layer was washed by water and dried over anhydrous Na_2SO_4. The solvent was removed under vacuum and the residue was purified by silica gel column chromatography (petroleum ether/CH_2Cl_2 = 1/1) to give compound **2** (88 mg) as a white solid in 70% yield. ^{1}H NMR (600 MHz, $CDCl_3$) δ = 8.83 (d, *J* = 7.8 Hz, 2H), 8.78 (d, *J* = 8.3 Hz, 2H), 8.68 (s, 2H), 8.20 (d, *J* = 7.4 Hz, 2H), 7.99 (s, 2H), 7.75 – 7.69 (m, 4H), 7.61 (t, *J* = 7.5 Hz, 2H), 7.43 (t, *J* = 7.5 Hz, 2H), 7.30 (d, *J* = 8.2 Hz, 2H), 3.45 (s, 6H), 2.50 (s, 1H), 2.49 (s, 5H). ppm. ^{13}C NMR (150 MHz, $CDCl_3$) δ = 167.09, 138.73, 135.83, 133.39, 132.55, 132.20, 132.13, 131.96, 131.34, 130.22, 129.95, 129.35, 127.04, 126.94, 126.65, 126.43, 125.75, 125.20, 123.15, 122.66, 52.29, 17.65. ppm. HRMS (ESI) (*m/z*): $[M+Na]^+$ calculated for $C_{44}H_{32}O_4Na$ 647.2193, found 647.2184.

Synthesis of Compound 3

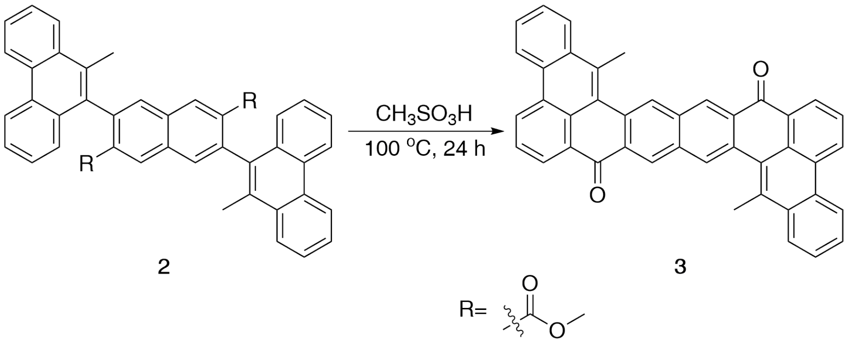

An oven-dried 25 mL Schlenk flask was charged with **2** (70 mg, 0.11 mmol) and methanesulphonic acid (6.0 mL) under argon. The mixture was degassed by three freeze-pump-thaw cycles and then it was refluxed at 100 °C for 24 hours. After cooling to room temperature, 30 mL water was added and stirred for 1 hour. The brown precipitate was filtered under vacuum, and washed several times with water, 50% $NaHCO_3$ aqueous solution, methanol and dried to give a reddish-brown powder (59 mg). Because of its very poor solubility, ^{1}H NMR, ^{13}C NMR with sufficient resolution could not be obtained and we directly proceeded to the next step.

Synthesis of DBOZ1

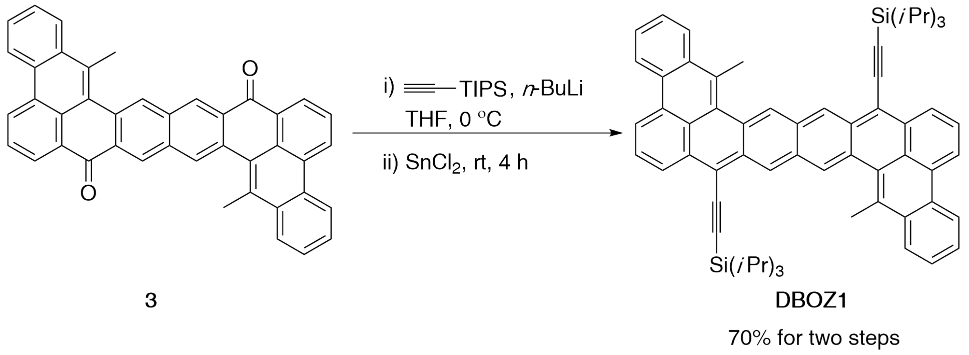

To a solution of triisopropylsilylacetylene (109 μL, 0.47 mmol) in dry tetrahydrofuran (4.0 mL) at 0 °C, *n*-BuLi (1.6 M in hexane, 0.26 mL, 0.40 mmol) was added dropwise and stirred for 1 hour. The diketone **3** (59 mg) was added to the solution under argon atmosphere and the mixture was stirred for 12 hours at room temperature until a clear solution was obtained. Then anhydrous $SnCl_2$ (330 mg, 1.72 mmol) was added under argon atmosphere. Upon addition, the color of the reaction mixture became blue instantaneously. This dark blue colored solution was then stirred for an additional 1

hour at room temperature. Upon completion of the reaction, solvent was removed and the residue was purified by silica gel column chromatography (petroleum ether/CH_2Cl_2=1:1) to give target compound DBOZ1 as a blue solid (69 mg, 70% yield for two steps). ^{13}C NMR couldn't be obtained due to the low solubility of the target molecule. ^{1}H NMR (600 MHz, TCE-d_2) δ = 8.71 (d, J = 8.2 Hz, 2H), 8.57 (d, J = 8.1 Hz, 2H), 8.29 (d, J = 8.0 Hz, 2H), 8.26 (d, J = 7.5 Hz, 2H), 8.17 (s, 2H), 7.79 – 7.76 (m, 2H), 7.75 – 7.70 (m, 4H), 7.61 (s, 2H), 3.20 (s, 6H), 1.31 (d, J = 4.5 Hz, 42H). ppm. HRMS (APCI) (m/z): $[M+H]^+$ calculated for $C_{64}H_{67}Si_2$ 891.4776, found 891.4777.

Synthesis of Compound 4

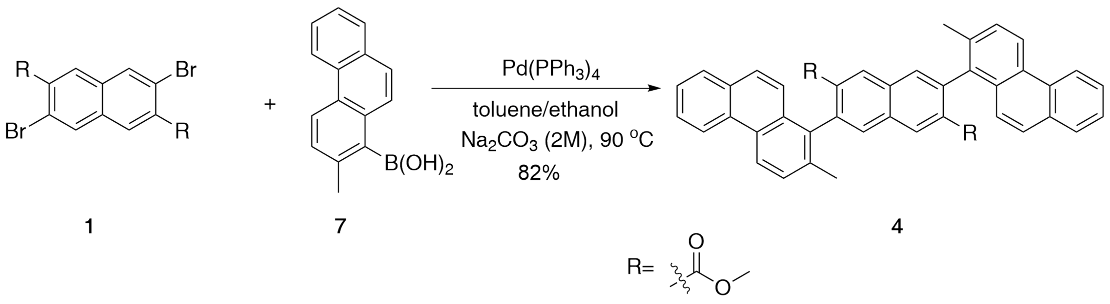

A mixture of dimethyl 3,7-dibromonaphthalene-2,6-dicarboxylate (80 mg, 0.20 mmol), compound **7** (236 mg, 1.0 mmol), $Pd(PPh_3)_4$ (24 mg, 0.02 mmol), toluene (2.5 mL), ethanol (0.3 mL), and Na_2CO_3 aqueous solution (2 M, 0.4 mL) were added and the mixture was further degassed by three freeze-pump-thaw cycles. The reaction was heated at 90 °C for 60 hours under argon atmosphere. After cooling to the room temperature, the mixture was poured into water and extracted with DCM. The organic layer was washed by water and dried over anhydrous Na_2SO_4. The solvent was removed under vacuum and the residue was purified by silica gel column chromatography (petroleum ether/CH_2Cl_2 = 2/1) to give compound **2** (103 mg) as a white solid in 82% yield. ^{1}H NMR (600 MHz, DMS0-d_6) δ = 8.88 (d, J = 8.3 Hz, 2H), 8.82 (d, J = 8.5 Hz, 2H), 8.78 (s, 2H), 8.18 (s, 2H), 7.91 (t, J = 8.1 Hz, 2H), 7.75 – 7.57 (m, 8H), 7.16 (d, J = 9.1 Hz, 1H), 7.13 (d, J = 9.2 Hz, 1H), 3.41 (s, 6H), 2.23 (s, 3H), 2.21 (s, 3H) ppm. ^{13}C NMR (150 MHz, DMS0-d_6) δ = 166.86, 138.18, 137.46, 134.42, 133.36, 132.02, 132.00, 131.21, 131.17, 130.86, 130.33, 129.20, 128.79, 128.29, 127.47, 127.19, 127.03, 124.71, 123.41, 122.66, 52.50, 21.05 ppm. HRMS (ESI) (m/z): $[M+Na]^+$ calculated for $C_{44}H_{32}O_4Na$ 647.2193, found 647.2202.

Synthesis of Compound 5

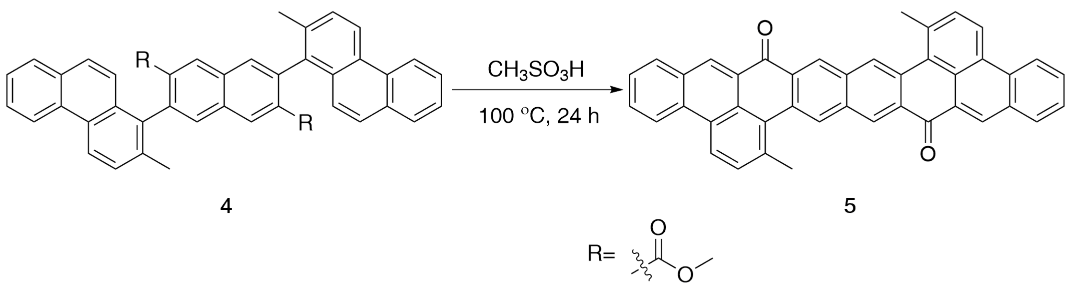

An oven-dried 25 mL Schlenk flask was charged with **4** (83 mg, 0.13 mmol) and methanesulphonic acid (8 mL) under argon. The mixture was degassed by three freeze-pump-thaw cycles and then it was refluxed at 100 °C for 24 hours. After cooling to room temperature, 40 mL water was added and stirred for 1 hour. The brown precipitate was filtered under vacuum, and washed several times with water, 50% $NaHCO_3$ aqueous solution, methanol and dried to give a reddish-brown powder (74 mg). Because of its very poor solubility, ^{1}H NMR, ^{13}C NMR with sufficient resolution could not be obtained and we directly proceeded to next step.

Synthesis of DBOZ2

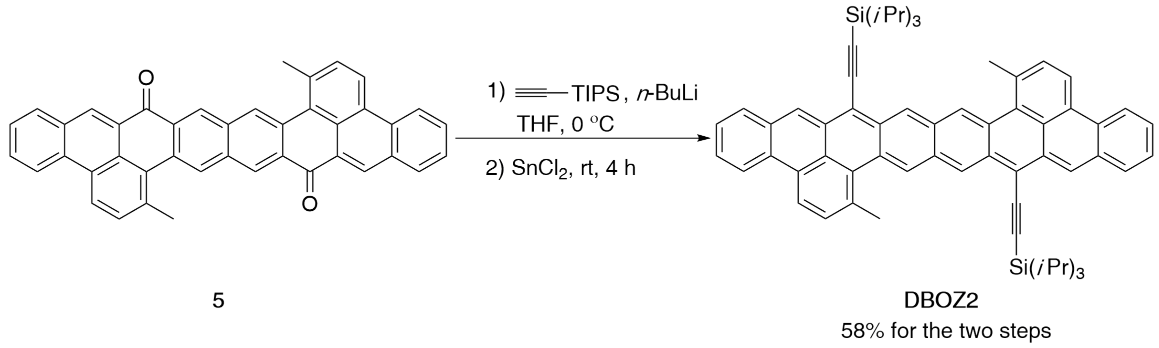

To a solution of triisopropylsilylacetylene (135 μL, 0.58 mmol) in dry tetrahydrofuran (5.0 mL) at 0 °C, *n*-BuLi (1.6 M in hexane, 0.32 mL, 0.49 mmol) was added dropwise and stirred for 1 hour. The diketone **5** (74 mg) was added to the solution under argon atmosphere and the mixture was stirred for 12 hours at room temperature until a clear solution was obtained. Then anhydrous $SnCl_2$ (219 mg, 1.15 mmol) was added under argon atmosphere. Upon addition, the color of the reaction mixture became blue instantaneously. This dark blue colored solution was then stirred for an additional 2 hours at room temperature. Upon completion of reaction, solvent was removed and the residue was purified by silica gel column chromatography (petroleum

ether/CH_2Cl_2=1:1) to give target compound DBOZ2 as a blue solid (69 mg, 58% yield for two steps). ^{13}C NMR couldn't be obtained due to the low solubility of the target molecule. ^{1}H NMR (600 MHz, $CDCl_3$) δ = 8.55 (d, J = 8.0 Hz, 4H), 8.43 (s, 2H), 8.21 (s, 2H), 7.95 (s, 2H), 7.83 (d, J = 7.4 Hz, 2H), 7.68 (d, J = 7.8 Hz, 2H), 7.58 – 7.53 (m, 4H), 3.05 (s, 6H), 1.35 (s, 42H). ppm. HRMS (APCI) (m/z): $[M+H]^+$ calculated for $C_{64}H_{67}Si_2$ 891.4776, found 891.4775.

2. Additional spectra and charts

2.1 HPLC analysis

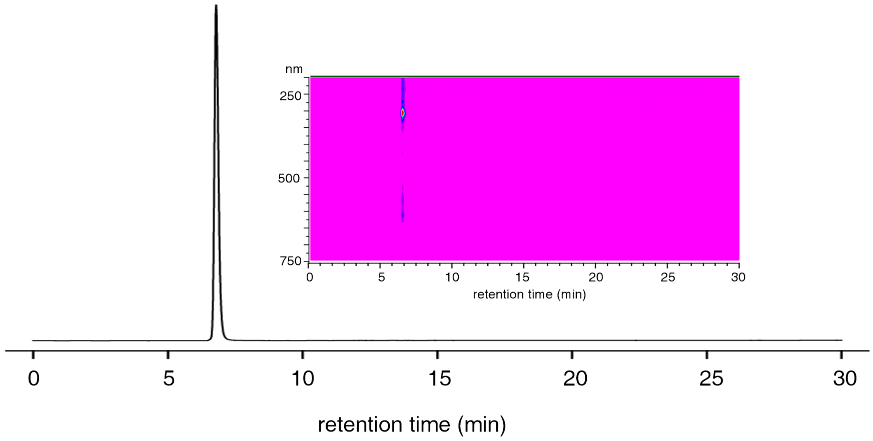

Figure S1. HPLC analysis for DBOZ1 with Cosmosil 5C18-MS-II column. Chromatographic conditions: flow rate = 0.5 mL/min, eluent = CH_2Cl_2 (90%)/ methanol (10%), column temperature = 25 °C, detection = 254 nm. Inset: 2D-HPLC chromatograms.

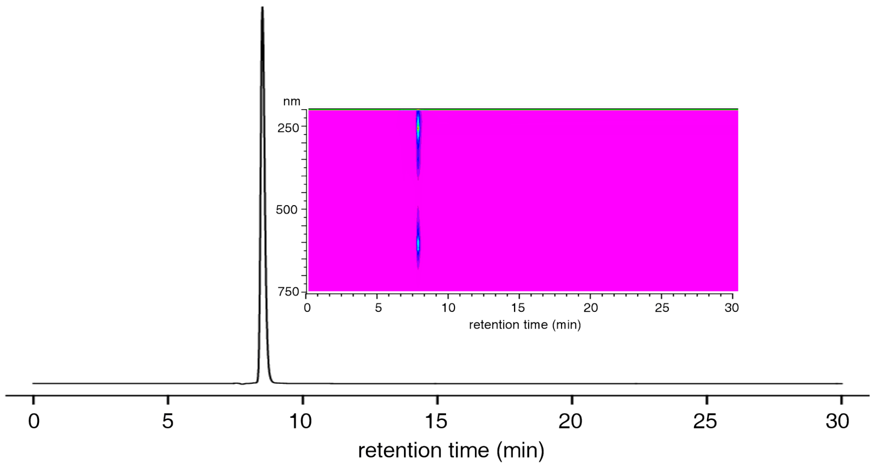

Figure S2. HPLC analysis for DBOZ2 with Cosmosil 5C18-MS-II column. Chromatographic conditions: flow rate = 0.4 mL/min, eluent = CH_2Cl_2 (80%)/ isopropanol (20%), column temperature = 25 °C, detection = 254 nm. Inset: 2D-HPLC chromatograms.

2.2 Solid state absorption and infrared spectra

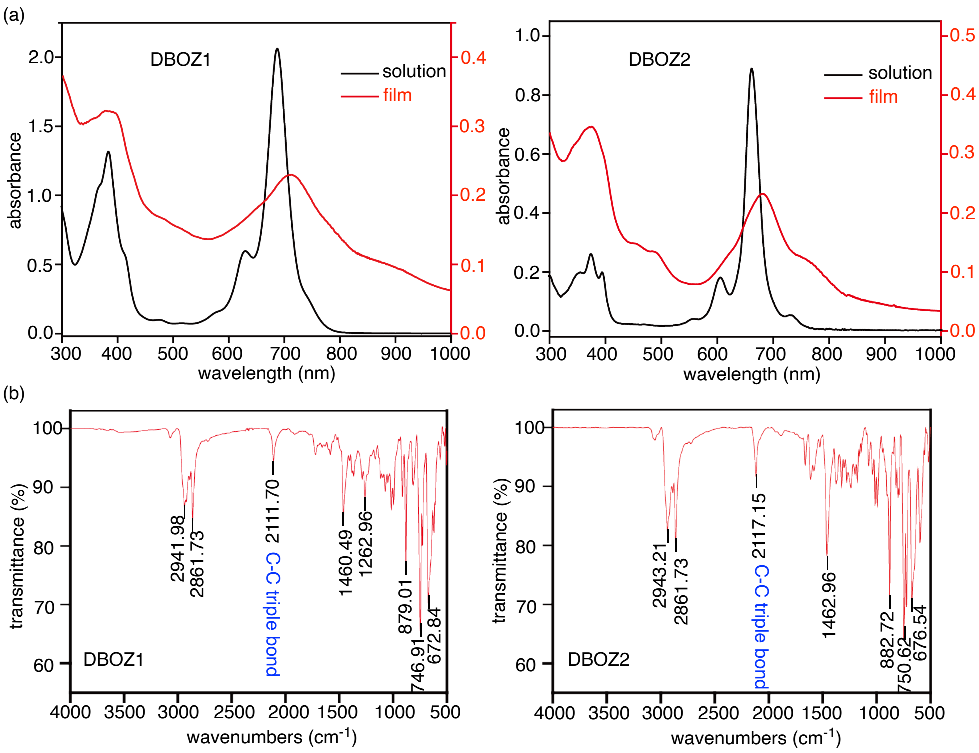

Figure S3 (a) Absorption spectra of DBOZ1 and DBOZ2 in toluene solution and in film. (b) Infrared spectra of the solid samples of DBOZ1 and DBOZ2.

2.3 Absorption intensity decay for stability test

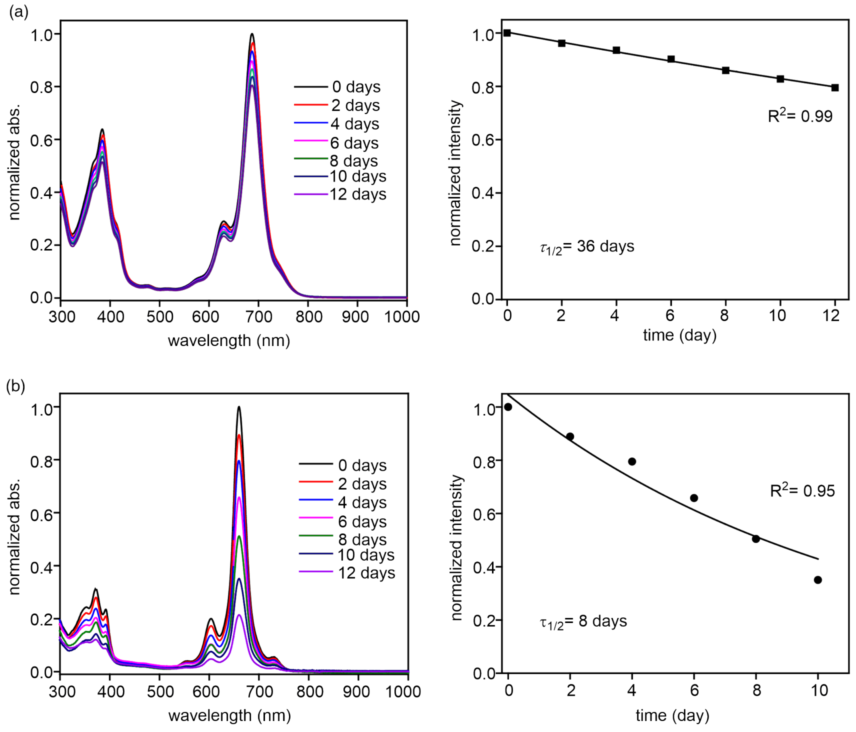

Figure S4. The UV-vis absorption spectral changes and fitting with first order kinetics of DBOZ1 (a) and DBOZ2 (b) under ambient light in toluene solutions.

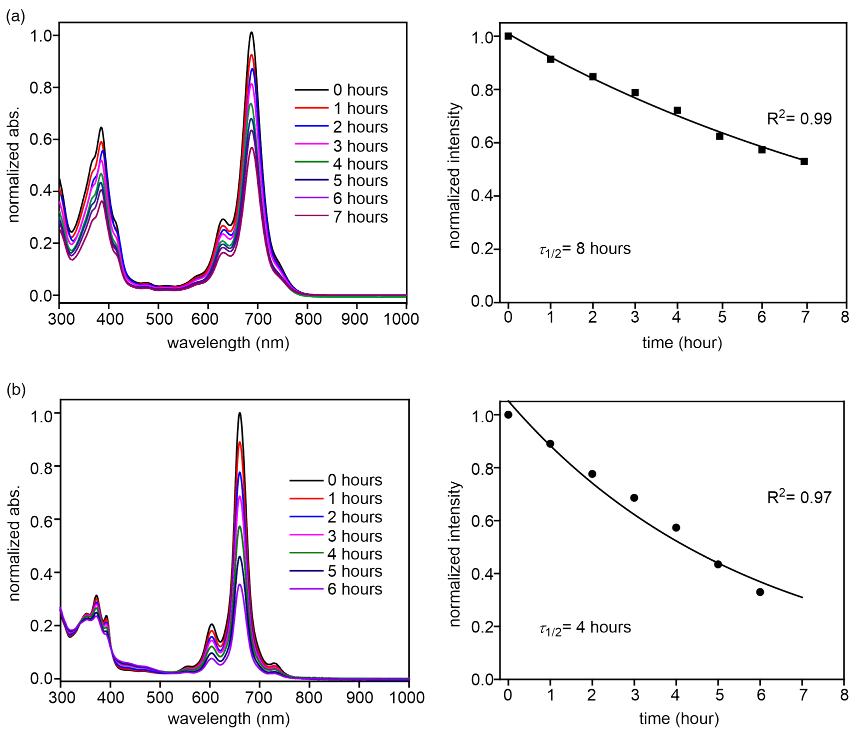

Figure S5. The UV-vis absorption spectral changes and fitting with first order kinetics of DBOZ1 (a) and DBOZ2 (b) under UV 254 nm light irradiation in toluene solutions.

2.4 TGA charts

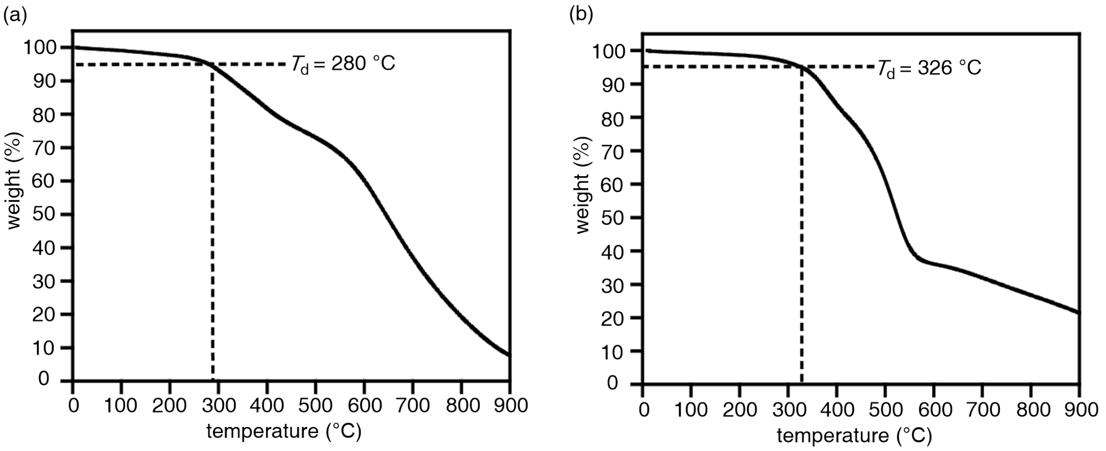

Figure S6. TGA curves of (a) DBOZ1 and (b) DBOZ2 at the heating rate of 10 °C /min under N_2 atmosphere.

2.5 VT NMR spectra

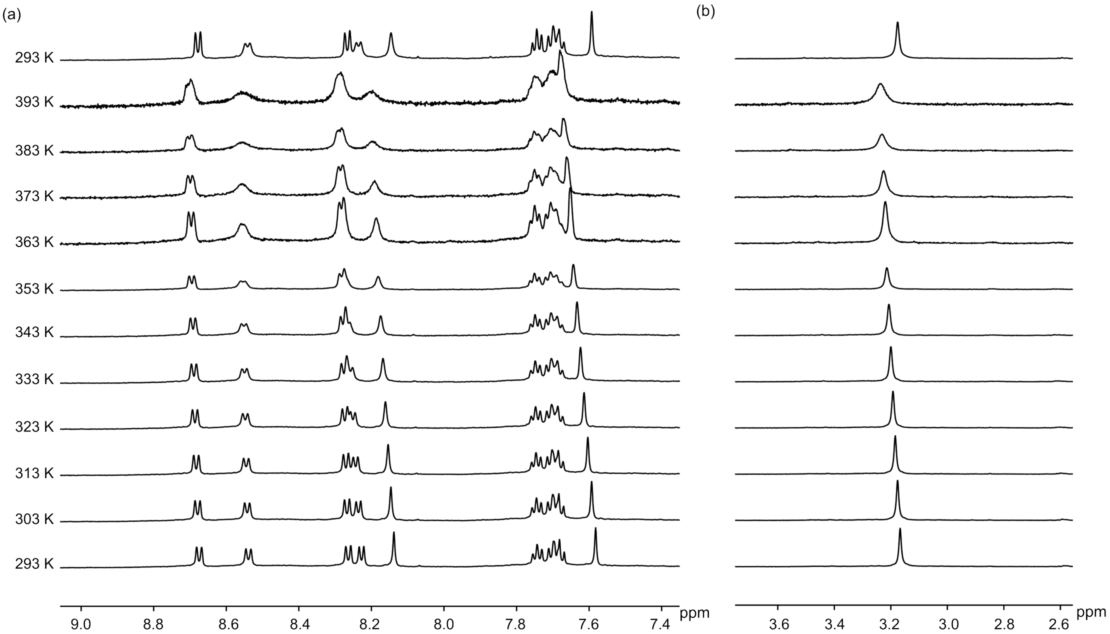

Figure S7. Variable-temperature ^{1}H NMR spectra of DBOZ1 at the (a) aromatic region and (b) region for methyl group (TCE-d_2, 600 MHz).

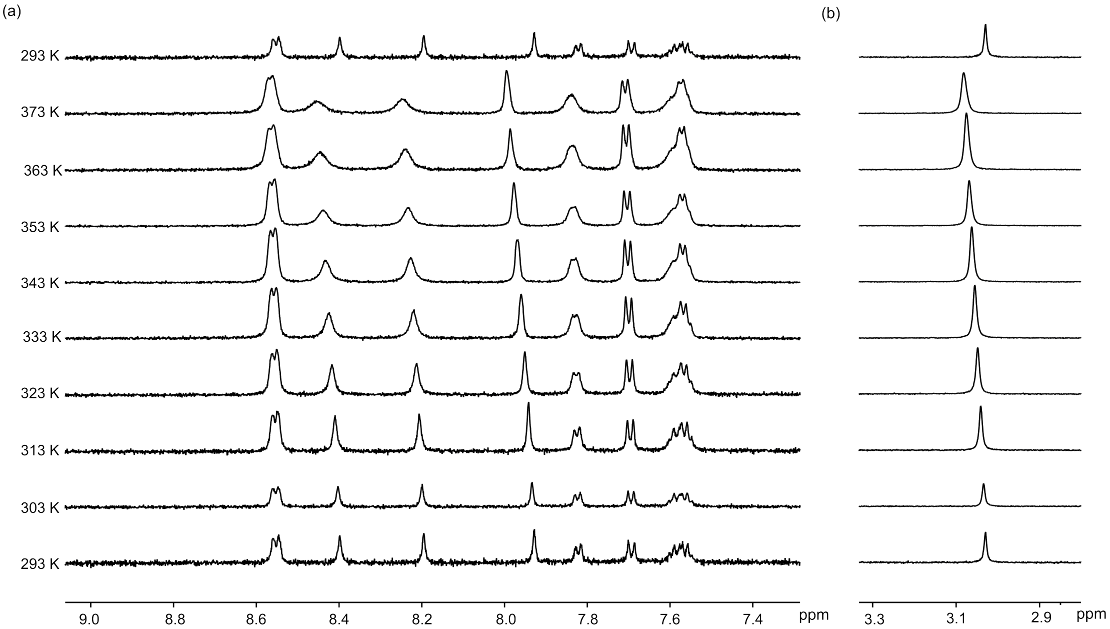

Figure S8. Variable-temperature ^{1}H NMR spectra of DBOZ2 at the (a) aromatic region and (b) region for methyl group (TCE-d_2, 600 MHz).

3. X-ray crystallographic data

Single crystals suitable for the X-ray analysis were obtained by slow diffusion of heptane to toluene for DBOZ1 and acetonitrile to *o*-dichlorobenzene for DBOZ2 at 0 ^{o}C. The single-crystal X-ray diffraction studies of them were carried out on the XtaLAB Synergy diffractometer using Cu Kα radiation (λ = 1.54184 Å). Both diffractometers are equipped with Hypix6000HE detector. The single crystal was mounted using oil on a nylon loop fixed on a goniometer head and immediately transferred to diffractometer. The obtained diffraction data were processed by the program suite CrysAlispro (Rigaku Oxford Diffraction, CrysAlisPro Software system, version 1.171.40_64.53, Rigaku Corporation, Oxford, UK, 2019). The structures were solved with dual-space method using SHELXT program[3] and refined with non-linear least square method using SHELXL program[4] embedded in the program suite OLEX2.[5] For more detailed information about diffraction data collection and refinement parameters, see Table S2 for DBOZ1 and Table S3 for DBOZ2. The crystallographic data were deposited in Cambridge Crystallographic Data Centre (CCDC 2090787 for DBOZ1, CCDC 2090790 for DBOZ2). The data can be achieved free of charge from www.ccdc.cam.ac.uk/data_request/cif.

Table S1. Crystal data and structure refinement for DBOZ1 at 293 K

Data deposition	CCDC 2090787
Empirical formula	$C_{64}H_{66}Si_2$
Formula weight	891.34
Temperature/K	293(2)
Crystal system	monoclinic
Space group	$P2_1/n$
Unit cell dimensions	a = 18.2848(5) Å
	b = 7.92270(10) Å
	c = 19.2800(6) Å
	α = 90 °
	β = 117.080(4) °
	γ = 90 °
Volume/ Å^3	2486.80(13)
Z	2
ρ_{calc}g/cm^3	1.190
μ/mm^{-1}	0.944
F(000)	956.0
Crystal size/mm^3	0.05 × 0.02 × 0.02
Radiation	Cu Kα (λ = 1.54784)
Theta range for data collection/°	5.526 to 153.18
Index ranges	$-20 \leq h \leq 22$, $-4 \leq k \leq 9$, $-24 \leq l \leq 24$
Reflections collected	16506
Independent reflections	4983 [R_{int} = 0.0498, R_{sigma} = 0.0486]
Data/restraints/parameters	4983/0/305
Goodness-of-fit on F^2	1.041
Final R indices [I>2σ(I)]	R_1 = 0.0515, wR_2 = 0.1384
R indices (all data)	R_1 = 0.0664, wR_2 = 0.1521
Largest diff. peak/hole / e Å^{-3}	0.35/-0.28

Table S2 Crystal data and structure refinement for DBOZ2 at 293 K.

Data deposition	CCDC 2090790
Empirical formula	$C_{64}H_{66}Si_2$
Formula weight	891.34
Temperature/K	293(2)
Crystal system	triclinic
Space group	*P*-1
Unit cell dimensions	a = 7.9997(2) Å
	b = 8.8382(2) Å
	c = 17.8954(3) Å
	α = 94.227(2) °
	β = 96.681(2) °
	γ = 94.149(2) °
Volume/ Å^3	1249.06(5)
Z	1
ρ_{calc}g/cm^3	1.185
μ/mm^{-1}	0.940
F(000)	487.0
Crystal size/mm^3	0.2 × 0.12 × 0.1
Radiation	Cu Kα (λ = 1.54784)
Theta range for data collection/°	9.988 to 149.352
Index ranges	$-9 \le h \le 10$, $-10 \le k \le 11$, $-21 \le l \le 21$
Reflections collected	14612
Independent reflections	4869 [R_{int} = 0.0400, R_{sigma} = 0.0357]
Data/restraints/parameters	4869/0/305
Goodness-of-fit on F^2	1.081
Final R indices [I>2σ(I)]	R_1 = 0.0712, wR_2 = 0.2005
R indices (all data)	R_1 = 0.0860, wR_2 = 0.2092
Largest diff. peak/hole / e Å^{-3}	0.46/-0.43

4. Theoretical calculations

The geometry optimizations and time-dependent DFT (TD-DFT) calculations were performed with the Gaussian09 program suite (Revision A.1).[6] The geometry optimizations were performed at UCAM-B3LYP/6-31G(d,p) level of theory. Time-dependent DFT (TD-DFT) calculations were performed at the UB3LYP/6-31G(d,p) level of theory. All optimized structures were confirmed to be true minima by vibrational analysis with no imaginary frequency. Diradical character indices (y_0) were determined by Yamaguchi scheme: y_0=1−[2T/(1+T^2)], where T equal to [(n_{HONO}-n_{LUNO})/2].[7] n_{HONO} and n_{LUNO} represent the occupation number of the highest occupied

natural orbital (HONO) and the occupation number of the lowest unoccupied natural orbital (LUNO), respectively, which were obtained by UCAM-B3LYP/6-31G(d,p) method. A molecule with y_0=0 indicates a closed-shell structure, whereas a molecule with 0<y_0<1 implies diradicaloid (diradical-like) structure.

4.1 TDDFT calculation

Table S3. Calculated electronic transitions for DBOZ1.

No.	wavelength (nm)	oscillator strength	major contributions
1	845.06	0.8087	HOMO→LUMO (98%)
2	768.49	0.0102	HOMO-1→LUMO (67%), HOMO→LUMO+1 (32%)
3	589.14	0.1949	HOMO→LUMO+1 (63%), HOMO-1→LUMO (35%)
4	513.51	0.0301	HOMO-3→LUMO (32%), HOMO-2→LUMO (44%), HOMO→LUMO+2 (8%), HOMO→LUMO+3 (6%)
5	472.56	0.0998	HOMO-6→LUMO (8%), HOMO-4→LUMO(22%), HOMO-5→LUMO (8%) HOMO-3→LUMO (17%), HOMO-2→LUMO (15%), HOMO→LUMO+3 (8%), HOMO→LUMO+4 (4%), HOMO→LUMO+6 (6%)
6	439.98	0.0078	HOMO-6→LUMO (11%), HOMO-5→LUMO (41%), HOMO-4→LUMO (6%), HOMO-2→LUMO (16%), HOMO→LUMO+3 (6%), HOMO→LUMO+4 (11%)
7	431.39	0.1997	HOMO-3→LUMO (8%), HOMO-2→LUMO (6%), HOMO→LUMO+1 (8%), HOMO-1→LUMO+1 (8%), HOMO→LUMO+2 (37%), HOMO→LUMO+3 (12%)
8	399.30	0.3870	HOMO-5→LUMO (5%),HOMO-4→LUMO (18%), HOMO→LUMO+3 (46%), HOMO→LUMO+2 (22%)

Table S4. Calculated electronic transitions for DBOZ2.

No.	wavelength (nm)	oscillator strength	major contributions
1	812.12	1.1682	HOMO→LUMO (98%)
2	721.02	0.0180	HOMO-1→LUMO (69%), HOMO→LUMO+1 (25%)
3	560.73	0.2200	HOMO→LUMO+1 (69%), HOMO-1→LUMO (28%)
4	528.21	0.0115	HOMO-2→LUMO (75%), HOMO→LUMO+2 (17%),
5	459.85	0.0005	HOMO-6→LUMO (4%), HOMO-2→LUMO(12%), HOMO-4→LUMO (60%) HOMO-1→LUMO+1 (14%)
6	459.29	0.0842	HOMO-6→LUMO (11%), HOMO-5→LUMO (11%), HOMO-4→LUMO (37%), HOMO-2→LUMO (4%), HOMO→LUMO+2 (5%), HOMO→LUMO+4 (7%), HOMO→LUMO+6 (6%)
7	440.25	0.0731	HOMO-5→LUMO (24%), HOMO-3→LUMO (14%), HOMO-2→LUMO (11%), HOMO→LUMO+2 (30%), HOMO→LUMO+5 (5%)
8	436.67	0.0785	HOMO-3→LUMO (48%),HOMO-2→LUMO (3%), HOMO-4→LUMO (2%), HOMO-6→LUMO (2%), HOMO→LUMO+2 (33%)

4.2 Diradical index

Table S5. Relative energies of optimized geometries, and diradical character index for substitute and unsubstituted dibenzooctazethrene derivatives at (U)CAM-B3LYP/6-31G(d,p) level.

compound	state	hartrees	$\Delta E_{S\text{-}T}$ (kcal/mol)	y_0
	Singlet Closed Shell	-3056.1757		
**DBOZ1**	Singlet Open Shell	-3056.1870	-5.73	0.34
	Triplet Open Shell	-3056.1779		
	Singlet Closed Shell	-3056.1836		
**DBOZ2**	Singlet Open Shell	-3056.1961	-5.32	0.37
	Triplet Open Shell	-3056.1876		

4.3 Transfer integrals and effective mass

All the calculations in this section were carried out based on the crystal structures. The reorganization energy and transfer integral were calculated at B3LYP/6-31G(d) and PBE0/6-31G(d) level, respectively.[8] Fermi's golden rule and Monte Carlo simulation were used to get the hole mobility.[9] The band structure and density of states were calculated using the projector augmented wave (PAW) method and the Perdew-Burke-Ernzerhof (PBE) exchange-correlation functional.[10] The plane wave cutoff is 400 eV and total energy convergence condition were set as 10^{-5} eV.

In the hopping model, the charge transport process could be viewed as intermolecular charge hopping. The charge transfer rates are from the Fermi's golden rule and reduced as,

$$k_{if} = \frac{V_{fi}^{\ 2}}{\hbar}\sqrt{\frac{\pi}{\lambda k_B T}}\exp(-\frac{\lambda}{4k_B T}) \qquad \text{Equation 1}$$

where V_{fi} is the electronic coupling between neighboring molecules, λ is the reorganization energy, k_B is the Boltzmann constant, and T is the temperature.

With the transfer rates, Mont Carlo random walk scheme was used to simulate the charge diffusion. Thereafter, the mobility can be calculated with the Einstein relationship and expressed by the following expression,

$$\mu = \frac{e}{k_B T} D \qquad \text{Equation 2}$$

Base on electronic band structure, the charge effective mass of the two molecular system can be calculated by,

$$m^* = \frac{\hbar^2}{\mathrm{d}E^2 / \mathrm{d}^2 k} \qquad \text{Equation 3}$$

5. OFET fabrication and characterization

5.1 Substrate preparation

SiO_2 (300 nm)/Si substrates were cleaned by sonification in deionized water, acetone and isopropanol for 10 min successively. the SiO_2/Si wafers were then tre ated by oxygen plasma at 80 W for 10 min followed by modification with octade cyltrichlorosilane (OTS) by a vapor phase method.

5.2 Crystal growth and characterization

Crystals of DBOZ1 and DBOZ2 were grown by drop casting. First, solutions of DBOZ1 (1 mg mL^{-1} in chlorobenzene) and DBOZ2 (0.2 mg mL^{-1} in chloroform) were prepared. Then, 20μL of the solutions were dropped on the OTS modified SiO_2 (300 nm)/Si wafers. After the solvent evaporated completely, crystals were obtained. The charge transport properties of crystals of DBOZ1 and DBOZ2 were investigated by constructing OFETs with a bottom-gate/top-contact configuration. OTS-modified SiO_2 (300 nm) was used as the gate dielectric layer, and silver (80 nm) was used as the source/drain electrode. The photoresponse of the OFETs based on DBOZ2 under 808 nm light irradiation was measured and photoresponse was observed in ambient air. The threshold voltage shifted to positive gate voltage and the current shifted upwards after illumination. At gate voltage of 6 V, the current increased by three orders of magnitude under illumination, which shows that DBOZ2 has the potential in infrared detection. Moreover, ambipolar transfer characteristics were performed in vacuum under dark conditions.

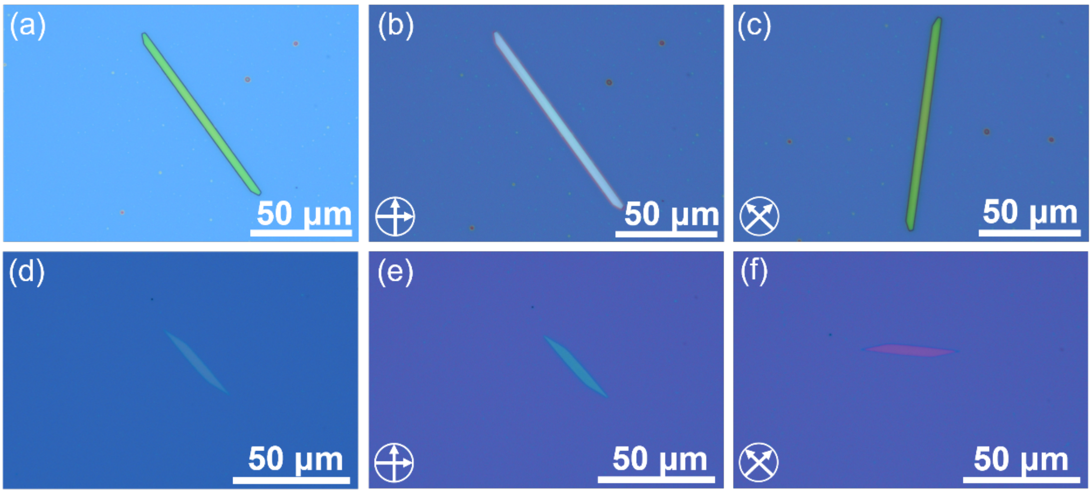

Figure S9. (a, d) OM images of crystals of DBOZ1 and DBOZ2; (b, c, e, f) POM images of crystals of DBOZ1 and DBOZ2.

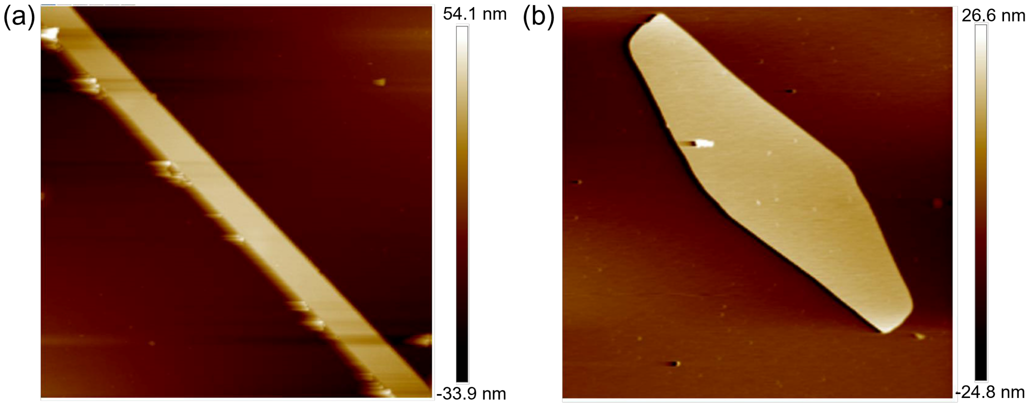

Figure S10. AFM images of crystals of (a) DBOZ1 and (b) DBOZ2.

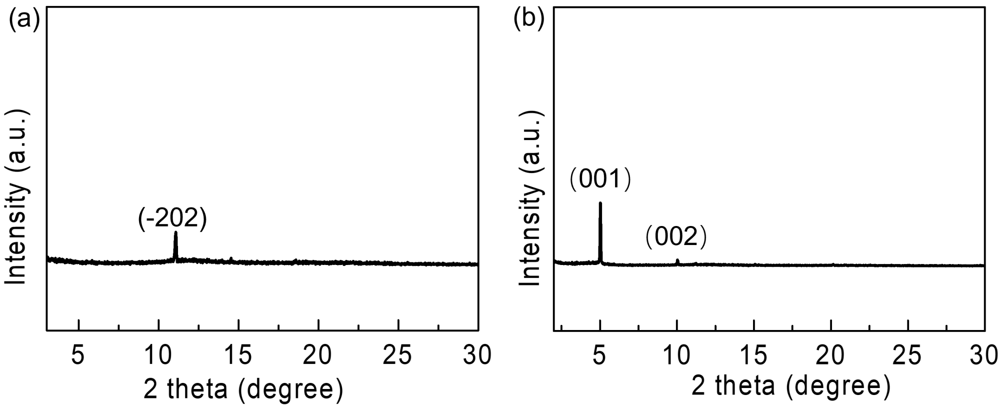

Figure S11. XRD patterns of crystals of (a) DBOZ1 and (b) DBOZ2. The sharp XRD peaks indicated that both microribbons of DBOZ1 and DBOZ2 exhibited highly ordered structure.

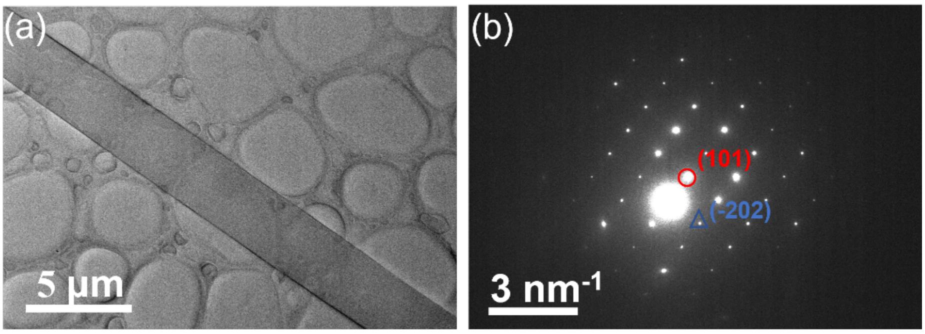

Figure S12. TEM (a) and the corresponding SAED pattern (b) of a crystal of DBOZ1.

5.3 Device characterization

OFETs with bottom-gate top-contact configurations were fabricated by gluing Ag (160 nm) layers on microribbons as the source and drain electrodes.[11] The mobility of the OFETs was calculated by the following equation: $I_{DS} = (W/2L)\mu C_i\ (V_{GS} - V_{th})^2$, where I_{DS} is the source-drain current, μ is the field-effect mobility, V_{th} is the threshold voltage, V_{GS} is the applied gate voltage, L is the channel length, W is the channel width and the C_i is capacitance per unit area (10 nF cm^{-2}).

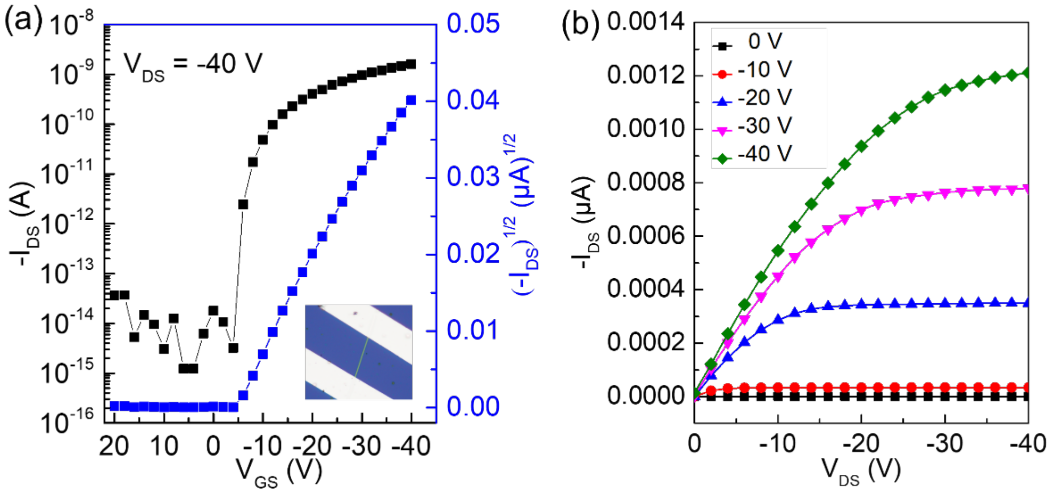

Figure S13. (a) Transfer and (b) output curves of OFETs based on crystals of DBOZ1 measured in air (channel length = 53.4 μm, channel width = 1.6 μm).

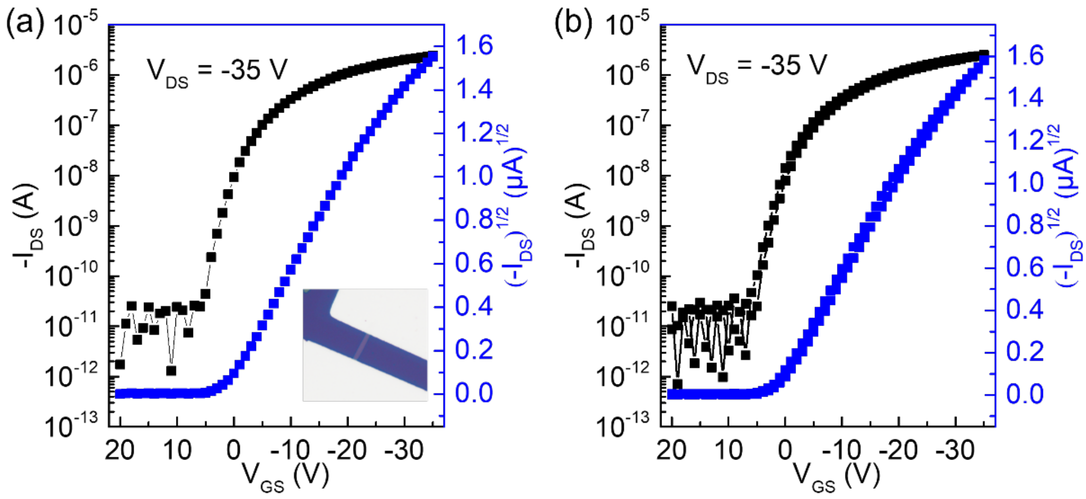

Figure S14. (a) Typical transfer and (b) hysteresis curves of the OFETs based on DBOZ2 measured in air with a maximum hole mobility as high as 3.5 $cm^2 V^{-1} s^{-1}$ (channel length = 15.4 μm, channel width = 2.0 μm).

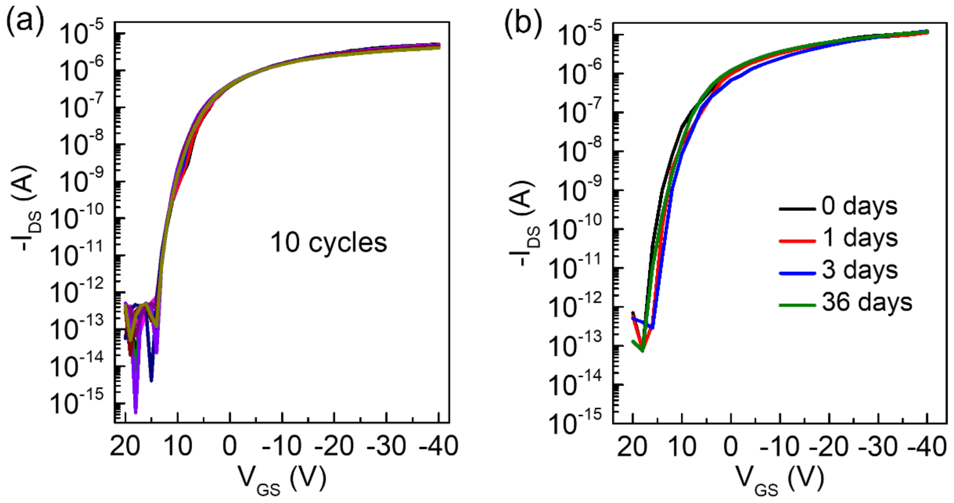

Figure S15. (a) Bias stress stability curves of OFETs based on crystals of DBOZ2 (channel length = 20.5 μm, channel width = 3.0 μm). (b) Transfer curves of OFETs based on crystals of DBOZ2 before and after storing in air for more than one month (channel length = 21.1 μm, channel width= 7.5 μm).

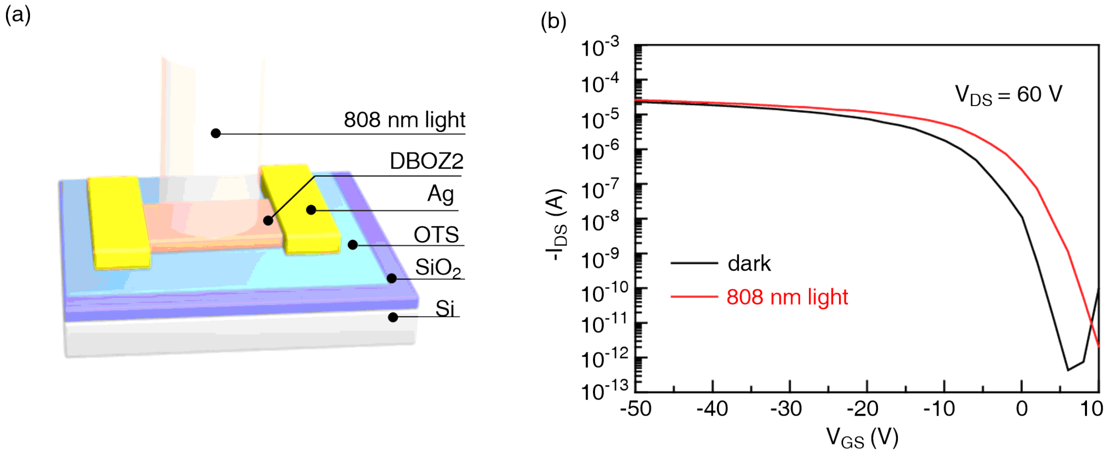

Figure S16. (a) Schematic diagram of organic phototransistors based on organic single crystal of DBOZ2. (b) Transfer curves of an organic phototransistor based on organic single crystal of DBOZ2 in the dark and under illumination.

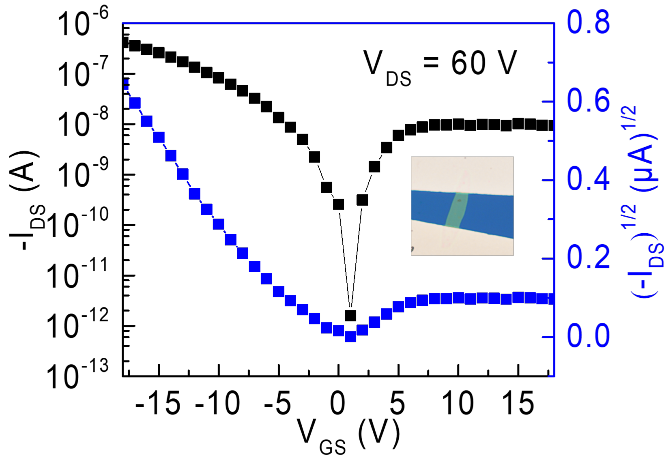

Figure S17. Typical ambipolar transfer characteristics of DBOZ2 under positive drain bias in vacuum.

5.4 Summary of singlet diradical semiconductors in the literature

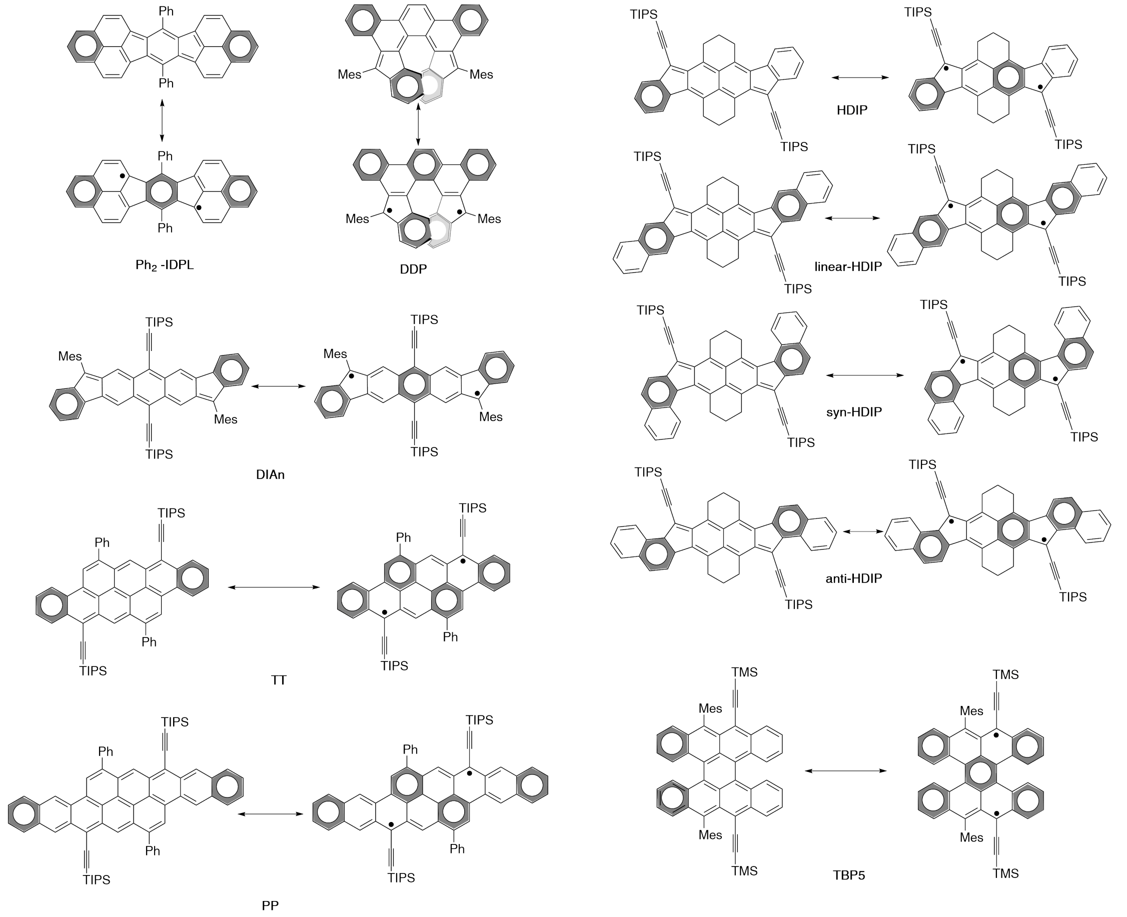

Figure S18 Chemical structures of selected singlet diradicals reported in the literature.

Table S6 Summary of device fabrication method and hole mobility of OFETs based on singlet diradicals

Materials	$\mu_{h, max}$ ($cm^2 V^{-1} s^{-1}$)	preparation method	test environment	published year	Ref.
**Ph_2-IDPL**	0.72	physical vapor transport	vacuum	2016	12
**DIAn**	4.5×10^{-2}	vapour deposition	air	2016	13
**DDP**	1.9×10^{-3}	spin-coating	nitrogen	2018	14
**TT**	0.77	drop-casting	nitrogen	2019	15
**PP**	1.4	drop-casting	nitrogen	2019	15
**HDIP**	0.4	drop-casting	nitrogen	2020	16
**linear-HDIP**	0.3	drop-casting	nitrogen	2020	16
**syn-HDIP**	1.3×10^{-3}	drop-casting	nitrogen	2020	16
**anti-HDIP**	0.2	drop-casting	nitrogen	2020	16
**TBP5**	1.2×10^{-2}	drop-casting	nitrogen	2021	17
**2,3:10,11-DBHZ**	0.15	layer-defining strategy	air	2021	2
**4,5:12,13-DBHZ**	0.017	layer-defining strategy	air	2021	2
**DBOZ2**	3.5	drop-casting	air	**This work**	

6. $^1H/^{13}C$NMR and MS spectra for new compounds

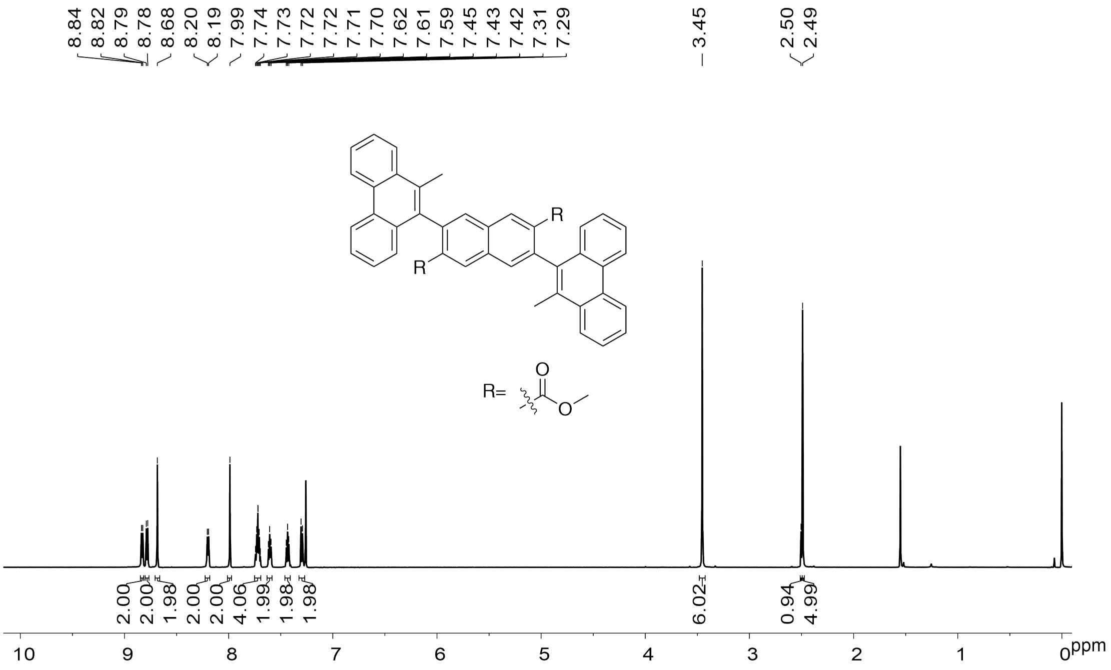

Figure S19 ^1H NMR spectrum of **2** in $CDCl_3$ (298 K, 600 MHz).

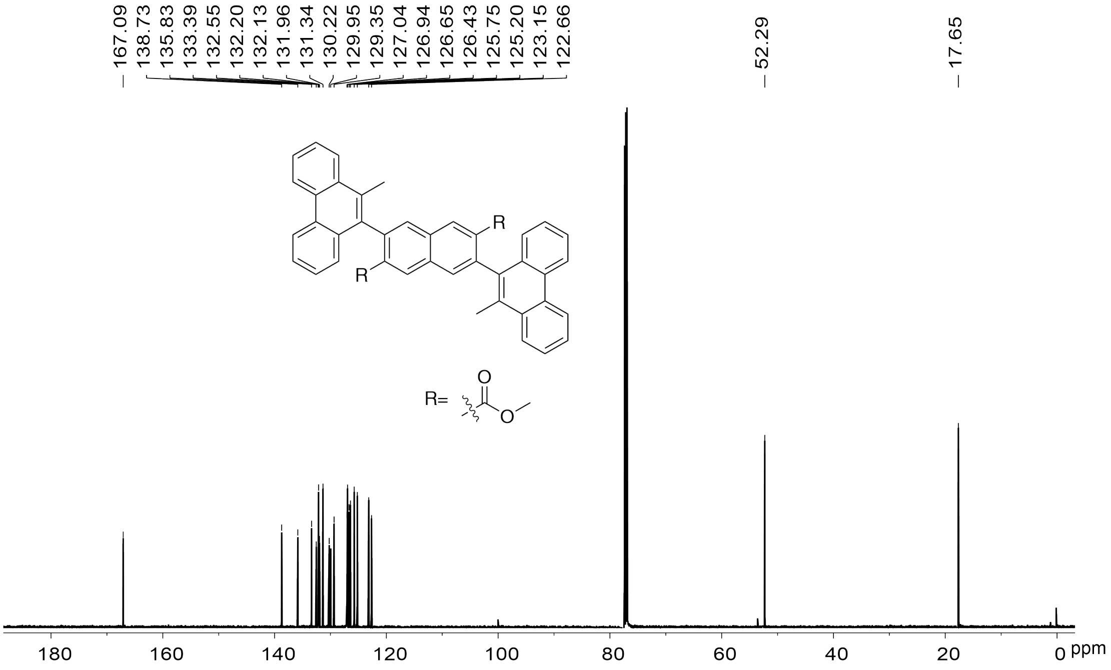

Figure S20. ^{13}C NMR spectrum of **2** in $CDCl_3$ (298 K, 150 MHz).

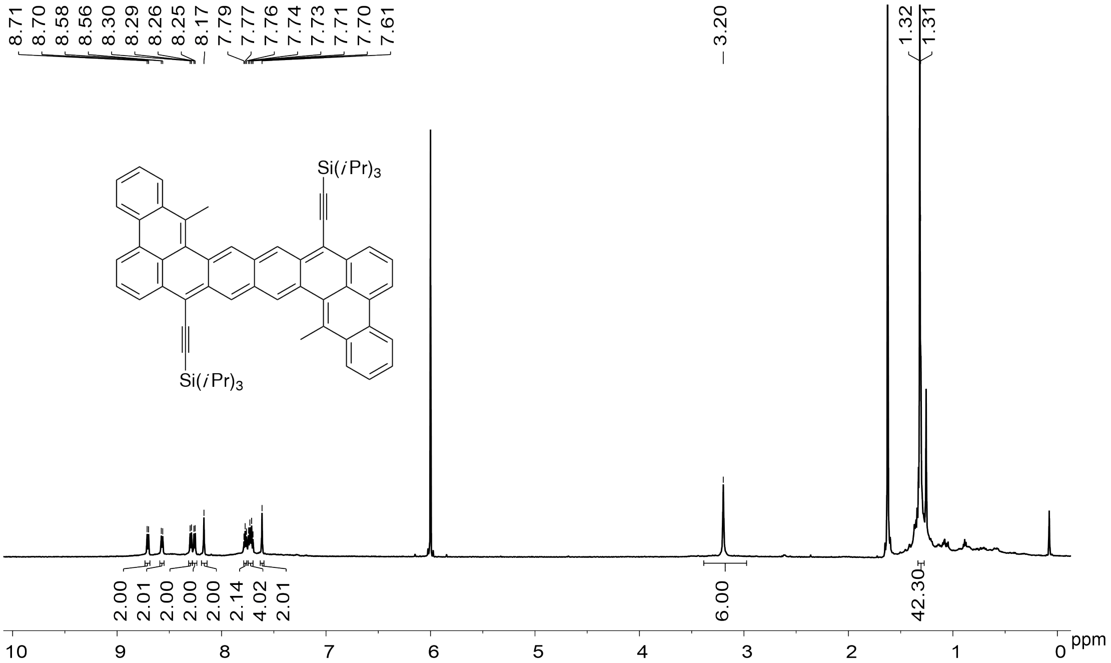

Figure S21. ^{1}H NMR spectrum of **DBOZ1** in TCE-d_2 (298 K, 600 MHz).

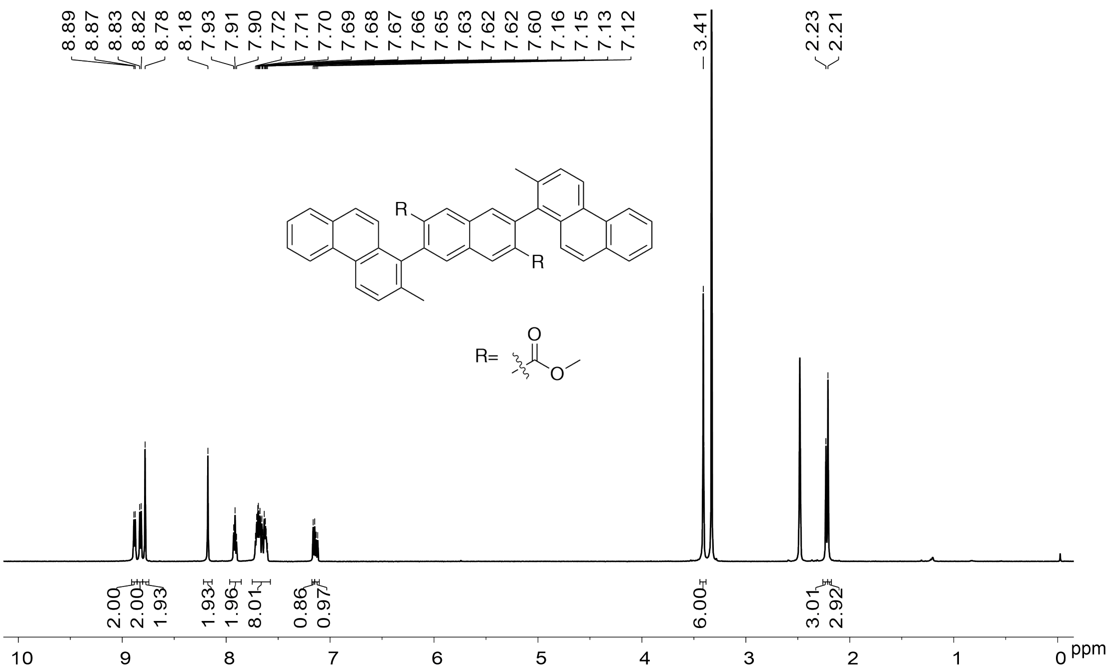

Figure S22. ^{1}H NMR spectrum of **4** in DMSO-d_6 (298 K, 600 MHz).

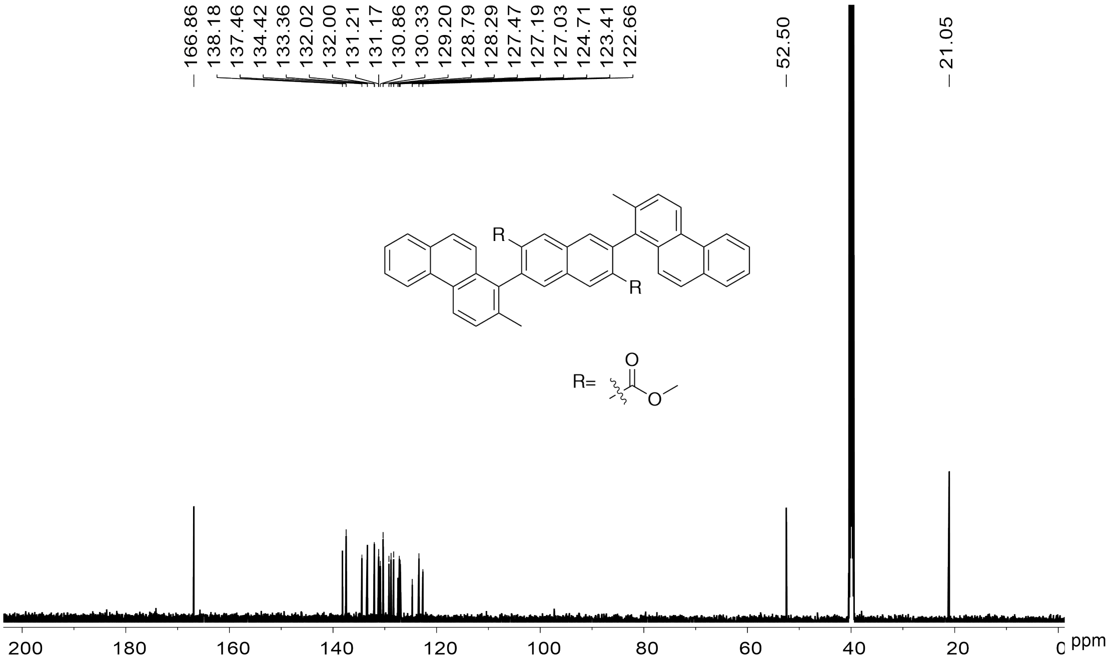

Figure S23. ^{13}C NMR spectrum of **4** in DMSO-d_6 (298 K, 150 MHz).

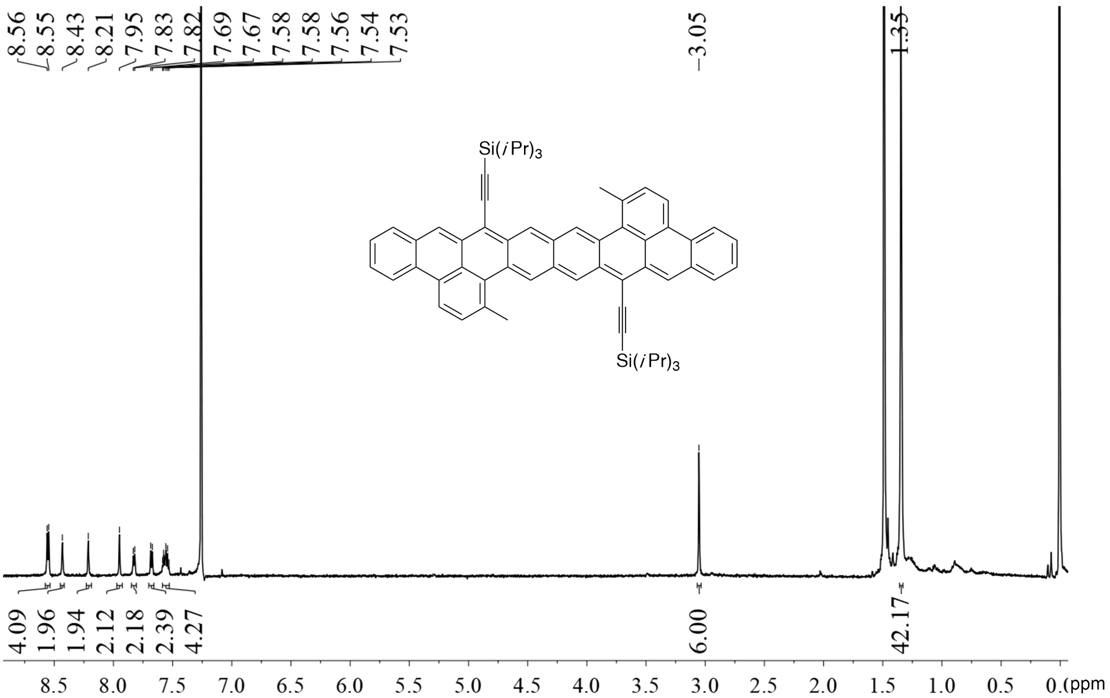

Figure S24. ^{1}H NMR spectrum of **DBOZ2** in $CDCl_3$ (298 K, 600 MHz).

Evaluation Spectra / Validation Formula

Meas. m/z	#	Formula	Score	m/z	err [mDa]	err [ppm]	mSigma	rdb	e⁻ Conf	N-Rule
647.2184	1	C 44 H 32 Na O 4	100.00	647.2193	0.9	1.4	93.9	28.5	even	ok

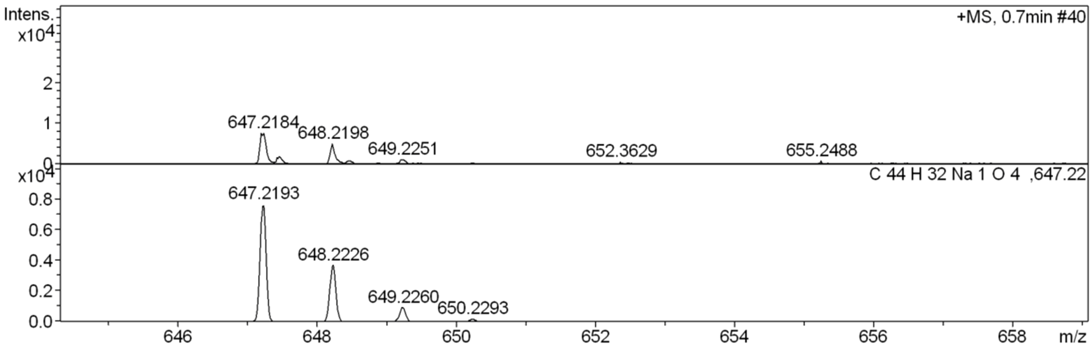

Figure S25. HR-ESI MS spectrum of **2**.

Evaluation Spectra / Validation Formula

Meas. m/z	#	Formula	Score	m/z	err [mDa]	err [ppm]	mSigma	rdb	e⁻ Conf	N-Rule
891.4777	1	C 64 H 67 Si 2	100.00	891.4776	-0.2	-0.2	69.6	33.5	even	ok

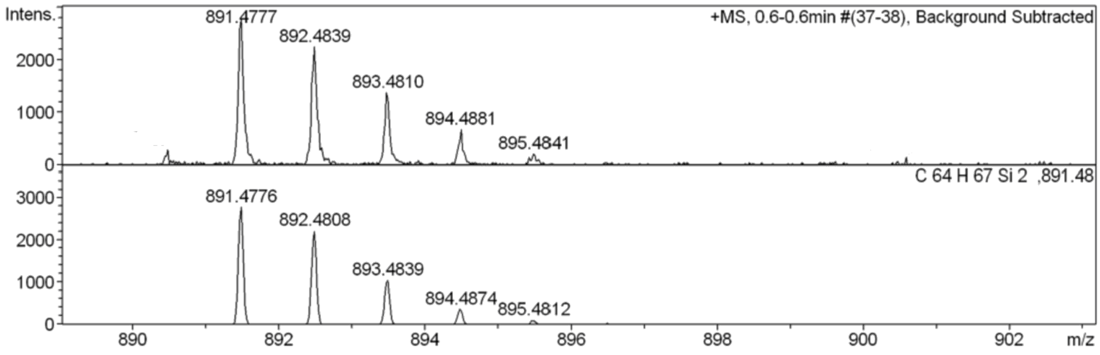

Figure S26. HR-APCI MS spectrum of DBOZ1.

Evaluation Spectra / Validation Formula

Meas. m/z	#	Formula	Score	m/z	err [mDa]	err [ppm]	mSigma	rdb	e⁻ Conf	N-Rule
647.2202	1	C 44 H 32 Na O 4	48.82	647.2193	-0.9	-1.4	105.3	28.5	even	ok

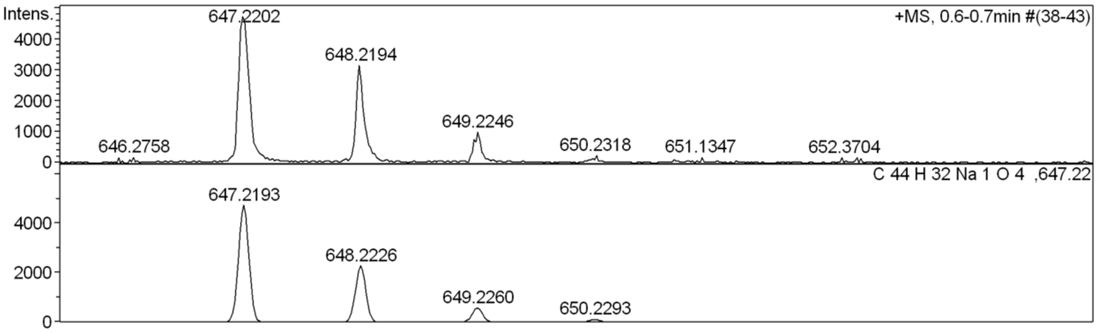

Figure S27. HR-ESI MS spectrum of **4**.

Evaluation Spectra / Validation Formula

Meas. m/z	#	Formula	Score	m/z	err [mDa]	err [ppm]	mSigma	rdb	e⁻ Conf	N-Rule
891.4775	1	C 64 H 67 Si 2	100.00	891.4776	0.1	0.1	69.3	33.5	even	ok

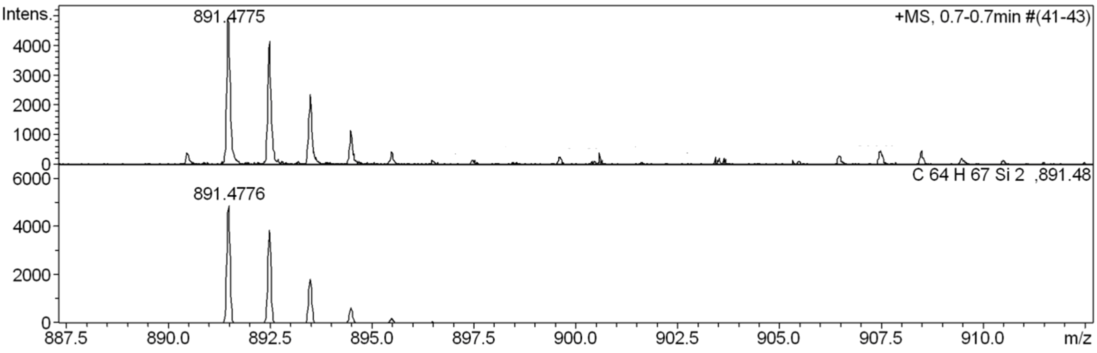

Figure S28. HR-APCI MS spectrum of DBOZ2.

7. References

1. J. Zhang, C. Yan, W. Wang, Y. Xiao, X. Lu, S. Barlow, T. C. Parker, X. Zhan and S. R. Marder, *Chem. Mater.,* 2018, **30**, 309-313.
2. C. Zong, X. Zhu, Z. Xu, L. Zhang, J. Xu, J. Guo, Q. Xiang, Z. Zeng, W. Hu, J. Wu, R. Li and Z. Sun, *Angew. Chem. Int. Ed.,* 2021, **60**, 16230-16236.
3. G. M. Sheldrick, *Acta Cryst. A,* 2015**, 71**, 3-8.
4. G. M. Sheldrick, *Acta Cryst. C,* 2015**, 71**, 3-8.
5. O. V. Dolomanov, L. J. Bourhis, R. J. Gildea, J. A. K. Howard and H. Puschmann, *J. Appl. Cryst.,* 2009**, 42**, 339-341.
6. Gaussian 09, Revision E.01. M. J. Frisch, G. W. Trucks, H. B. Schlegel, G. E. Scuseria, M. A. Robb, J. R. Cheeseman, G. Scalmani, V. Barone, B. Mennucci, G. A. Petersson, H. Nakatsuji, M. Caricato, X. Li, H. P. Hratchian, A. F. Izmaylov, J. Bloino, G. Zheng, J. L. Sonnenberg, M. Hada, M. Ehara, K. Toyota, R. Fukuda, J. Hasegawa, M. Ishida, T. Nakajima, Y. Honda, O. Kitao, H. Nakai, T. Vreven, J. A. Montgomery, Jr., J. E. Peralta, F. Ogliaro, M. Bearpark, J. J. Heyd, E. Brothers, K. N. Kudin, V. N. Staroverov, T. Keith, R. Kobayashi, J. Normand, K. Raghavachari, A. Rendell, J. C. Burant, S. S. Iyengar, J. Tomasi, M. Cossi, N. Rega, J. M. Millam, M. Klene, J. E. Knox, J. B. Cross, V. Bakken, C. Adamo, J. Jaramillo, R. Gomperts, R. E. Stratmann, O. Yazyev, A. J. Austin, R. Cammi, C. Pomelli, J. W. Ochterski, R. L. Martin, K. Morokuma, V. G. Zakrzewski, G. A. Voth, P. Salvador, J. J. Dannenberg, S. Dapprich, A. D. Daniels, O. Farkas, J. B. Foresman, J. V. Ortiz, J. Cioslowski, and D. J. Fox, Gaussian, Inc., Wallingford CT, 2013.
7. (a) S. Yamanaka, M. Okumura, M. Nakano and K. Yamaguchi, *J. Mol. Struct.,* 1994, **310**, 205-218; (b) K. Kamada, K. Ohta, A. Shimizu, T. Kubo, R. Kishi, H. Takahashi, E. Botek, B. Champagne and M. Nakano, *J. Phys. Chem. Lett.,* 2010, **1**, 937-940.
8. Gaussian 16, Revision A.03, M. J. Frisch, G. W. Trucks, H. B. Schlegel, G. E. Scuseria, M. A. Robb, J. R. Cheeseman, G. Scalmani, V. Barone, G. A. Petersson, H. Nakatsuji, X. Li, M. Caricato, A. V. Marenich, J. Bloino, B. G. Janesko, R. Gomperts, B. Mennucci, H. P. Hratchian, J. V. Ortiz, A. F. Izmaylov, J. L. Sonnenberg, D. Williams-Young, F. Ding, F. Lipparini, F. Egidi, J. Goings, B. Peng, A. Petrone, T. Henderson, D. Ranasinghe, V. G. Zakrzewski, J. Gao, N. Rega, G. Zheng, W. Liang, M. Hada, M. Ehara, K. Toyota, R. Fukuda, J. Hasegawa, M. Ishida, T. Nakajima, Y. Honda, O. Kitao, H. Nakai, T. Vreven, K. Throssell, J. A., Jr. Montgomery, J. E. Peralta, F. Ogliaro, M. J. Bearpark, J. J. Heyd, E. N. Brothers, K. N. Kudin, V. N. Staroverov, T. A. Keith, R. Kobayashi, J. Normand, K. Raghavachari, A. P. Rendell, J. C. Burant, S. S. Iyengar, J. Tomasi, M. Cossi, J. M. Millam, M. Klene, C. Adamo, R. Cammi, J. W. Ochterski, R. L. Martin, K. Morokuma, Ö. Farkas, J. B. Foresman, D. J. Fox, Gaussian, Inc., Wallingford CT, 2016.
9. Y. Niu, W. Li, Q. Peng, H. Geng, Y. Yi, L. Wang, G. Nan, D. Wang and Z. Shuai, *Mol. Phys.,* 2018, **116**, 1078-1090.
10. (a) G. Kresse and J. Furthmuller, *Phys. Rev. B,* 1996, **54**, 11169-11186; (b) G. Kresse and J. Furthmuller, *Comp. Mater. Sci.,* 1996, **6**, 15-50; (c) J. P. Perdew, K.

Burke and M. Ernzerhof, *Phys. Rev. Lett.,* 1996, **77**, 3865-3868.
11. Q. Tang, L. Jiang, Y. Tong, H. Li, Y. Liu, Z. Wang, W. Hu, Y. Liu and D. Zhu, *Adv. Mater.,* 2008**, 20**, 2947-2951.
12. H. Koike, M. Chikamatsu, R. Azumi, J. Tsutsumi, K. Ogawa, W. Yamane, T. Nishiuchi, T. Kubo, T. Hasegawa and K. Kanai, *Adv. Funct. Mater.* 2016, **26**, 277-283.
13. G. E. Rudebusch, J. L. Zafra, K. Jorner, K. Fukuda, J. L. Marshall, I. Arrechea-Marcos, G. L. Espejo, R. P. Ortiz, C. J. Gomez-Garcia, L. N. Zakharov, M. Nakano, H. Ottosson, J. Casado and M. M. Haley, *Nat. Chem.,* 2016, **8**, 753-759.
14. Y.-C. Hsieh, C.-F.Wu, Y.-T. Chen, C.-T. Fang, C.-S. Wang, C.-H. Li, L.-Y. Chen, M.-J. Cheng, C.-C. Chueh, P.-T. Chou and Y.-T. Wu, *J. Am. Chem. Soc.,* 2018, **140**, 14357-14366.
15. T. Jousselin-Oba, M. Mamada, J. Marrot, A. Maignan, C. Adachi, A. Yassar and M. Frigoli, *J. Am. Chem. Soc.,* 2019, **141**, 9373-9381.
16. T. Jousselin-Oba, M. Mamada, A. Okazawa, J. Marrot, T. Ishida, C. Adachi, A. Yassar and M. Frigoli, *Chem. Sci.,* 2020, **11**, 12194-12205.
17. M. Mamada, R. Nakamura and C. Adachi, *Chem. Sci.,* 2021, **12**, 552-558.
